# Optimal aerobic exercise dose for pain relief in fibromyalgia syndrome: a systematic review and meta-analysis

**DOI:** 10.3389/fphys.2025.1693735

**Published:** 2025-12-08

**Authors:** Yiqi Li, Yuan Yuan, Lijun Hua, Linghua Ran, Bopeng Qiu, Yong Yang, Lin Zhang

**Affiliations:** 1 Physical Education and Sports Science, Harbin Normal University School, Harbin, China; 2 Department of Sport Industry Studies, Yonsei University, Seoul, Republic of Korea; 3 Physical Education and Training, Harbin Sport University, Harbin, China; 4 School of Strength and Conditioning, Beijing Sport University, Beijing, China; 5 Laboratory of Kinesiology and Rehabilitation, School of Physical Education and Sport, Chaohu University, Hefei, China; 6 Department of Rehabilitation, West China Hospital Sichuan University Jintang Hospital, Chengdu, China

**Keywords:** fibromyalgia syndrome, pain, exercise, dose-response, meta-analysis, systematic review

## Abstract

**Background:**

Fibromyalgia syndrome (FMS) is a chronic disorder marked by widespread pain. While aerobic exercise (AE) is widely recommended, its isolated effect and the optimal regimen and dose remain unclear. This study aimed to clarify the independent effect of pure AE on pain in FMS and identify the most effective exercise parameters and dose-response relationship.

**Methods:**

We systematically searched PubMed, Cochrane, Embase, and Web of Science up to June 2025 for randomized controlled trials (RCTs) examining AE’s effect on FMS pain. Subgroup analyses were performed by age, baseline pain level, exercise type, and protocol characteristics. Standardized mean differences (SMD) with 95% confidence intervals (CIs) were calculated, and dose–response relationships were explored.

**Results:**

Fourteen RCTs with 777 participants were included. AE significantly reduced pain compared with controls [SMD = −1.07; 95% CI: −1.57 to −0.57], especially in patients aged 45–60 years and those with chronic severe pain. The most effective intervention was moderate-intensity, water-based AE performed for 60 min per session, 1–2 times weekly, over 12–16 weeks. Dose–response analysis identified an optimal dose of 470 MET-min/week [SMD = −1.71; 95% CrI: −1.90 to −1.14], with an effective range of 75–750 MET-min/week.

**Conclusion:**

This meta-analysis confirms the efficacy of AE in reducing pain in FMS. AE significantly alleviates pain in fibromyalgia, with moderate-intensity, water-based, showing the most effective results. These findings provide strong evidence for incorporating AE as a key non-pharmacological strategy in the management of fibromyalgia, especially for patients with more severe pain. The identified optimal exercise parameters offer clear guidance for clinical practice, supporting the use of tailored exercise regimens in patient care.

**Systematic Review Registration:**

https://www.crd.york.ac.uk/PROSPERO/view/, identifier CRD420251086595.

## Graphical Abstract

**Figure:**
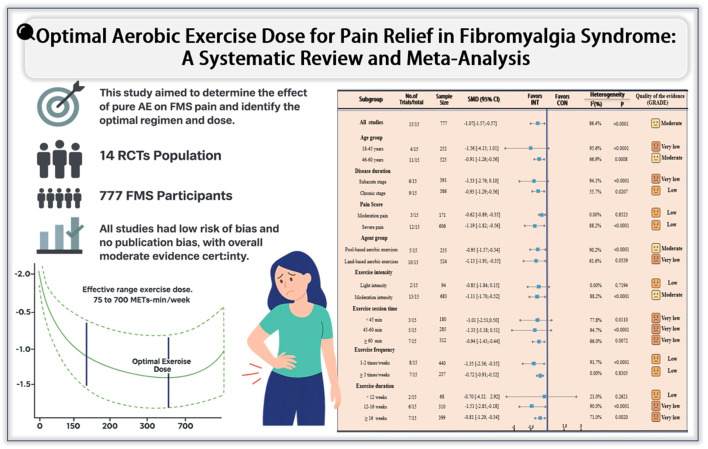

## 1 Introduction

Fibromyalgia syndrome (FMS) is a clinical condition primarily characterized by widespread, chronic musculoskeletal pain, often accompanied by fatigue, sleep disturbances, cognitive impairment, and psychological issues (Berwick et al., 2022). Although the precise pathophysiology of FMS remains unclear, it is widely believed to involve abnormalities in central pain processing, dysregulation of the neuroendocrine system, and various psychosocial factors (Fitzcharles et al., 2021). The global prevalence of FMS is estimated to be approximately 2%–4%, with a significantly higher incidence in women compared to men (Häuser et al., 2014). The widespread nature of this condition, particularly its association with chronic pain, means FMS profoundly impacts patients’ quality of life, work capacity, and social functioning (Annemans et al., 2009; Sarzi-Puttini et al., 2020).

In recent years, exercise interventions have received increasing attention as a low-risk and effective non-pharmacological treatment for addressing the challenges posed by FMS (Siracusa et al., 2021; Mannerkorpi, 2005). Given the limited efficacy and potential adverse effects of pharmacological therapies, non-pharmacological approaches have become a cornerstone in the management of FMS (Andrade et al., 2020; Wilson et al., 2012). Among these, aerobic exercise (AE) has emerged as a particularly promising intervention due to its safety, accessibility, and wide-ranging benefits (Andrade et al., 2019; Britto et al., 2020). The randomized controlled trials (RCTs) by Letieri et al. (2013) and Espí-López et al. (2016) have demonstrated that AE can effectively alleviate pain, improve physical function, and enhance overall health in patients with FMS (Letieri et al., 2013; Espí-López et al., 2016). The physiological mechanisms underlying these benefits may include enhanced central pain modulation, improved cardiovascular function, and reduced systemic inflammation (Carvalho et al., 2020; De Medeiros et al., 2020).

Although previous systematic reviews support AE for FMS pain management, methodological limitations persist (Häuser et al., 2010). Casanova-Rodríguez et al. (2025) demonstrated efficacy but pooled AE with other modalities, obscuring its specific effects (Casanova-Rodríguez et al., 2025). While Núñez-Cortés et al. (2025) addressed this by restricting to aerobic-exercise-only interventions, their within-group comparison design failed to control for placebo effects, and dose definition relied solely on duration without intensity parameters (Núñez-Cortés et al., 2025). More importantly, previous meta-analyses have attempted to quantify exercise intensity using metabolic equivalents (METs) (Yuan et al., 2024; Wang et al., 2025), but no studies have been conducted to explore the dose-response relationship of AE in relieving pain in FMS patients. Consequently, the optimal dosing parameters for AE—particularly intensity quantified by METs—remain poorly defined for pain management in fibromyalgia syndrome.

Based on this evidence gap, we conducted a systematic review and meta-analysis to investigate the impact of AE on pain alleviation in FMS. Our primary objective was to evaluate the independent net therapeutic effect of AE-only interventions on fibromyalgia pain, while controlling for confounding factors such as mixed interventions and placebo effects. Additionally, we aimed to identify optimal exercise protocols through meta-regression analyses that examined potential moderators, including age, baseline pain, disease duration, exercise type, program length, frequency, and exercise intensity. Lastly, we sought to establish dose-response relationships using METs metrics to develop precision prescription frameworks that can be translated into clinical practice for the management of FMS.

## 2 Materials and methods

This systematic review was conducted according to the guidelines of the Cochrane Collaboration and the Preferred Reporting Items for Systematic Reviews and Meta-Analyses (PRISMA) statement (Page et al., 2021).

### 2.1 Search strategy

To ensure comprehensive coverage of studies on AE interventions related to FMS, a systematic literature search was conducted by two independent reviewers between 23 February 2023, and 1 June 2023. Searches were performed in major electronic databases including PubMed, the Cochrane Library, Embase, and Web of Science, covering all relevant literature published up to 1 June 2025. In addition, the reference lists of included articles and previously published systematic reviews were manually screened to identify potentially relevant studies that may have been missed during the database search. All records were independently screened by the reviewers, and studies unrelated to the research objectives were excluded, as detailed in Supplementary Data 1.

### 2.2 Inclusion and exclusion criteria

The criteria for inclusion and exclusion were determined according to the PICOS framework (Participants, Interventions, Comparators, Outcomes, and Study design) (Schardt et al., 2007), as presented in Table 1. Using these predefined standards, two reviewers independently assessed potentially eligible articles at the title, abstract, and full-text levels to confirm their suitability.

**TABLE 1:** PICOS framework.

Items	Exclusion criteria	Inclusion criteria
Population (P)	Patients diagnosed with FMS based on the American College of Rheumatology (ACR) classification criteria and aged 35 years or older	The study population was not evaluated as FMS patients at baseline
Intervention (I)	AE interventions (e.g., walking, swimming, cycling)	Non-aerobic exercise interventions (e.g., resistance training, stretching)
Comparator (C)	Comparisons with no exercise intervention or standard care (e.g., usual care, wait-list)	Studies with no control group (e.g., single-arm trials)
Outcome (O)	Studies should include the outcome measure of interest: Pain(Visual Analogue Scale, Functional Activity Score)	Pain was not assessed as an outcome based on score changes between baseline and follow-up
Study Design (S)	Published RCTs were included in the study design	Non-randomized studies (e.g., cohort studies, case-control studies, observational studies)

FMS: fibromyalgia syndrome; ACR: american college of rheumatology; AE: aerobic exercise; RCTs: Randomized controlled trials.

### 2.3 Data extraction

Data extraction was performed independently by two reviewers. The extracted information included the following components: i) Study characteristics: first author’s last name, year of publication, and sample size; ii) Intervention variables: type of AE (e.g., aquatic or land-based), as a categorical variable, and continuous variables such as intervention duration (weeks), session duration (minutes), frequency (sessions per week), and total exercise time per week (minutes); iii) Participant characteristics: mean age and baseline pain level; iv) Primary outcome: pre- and post-intervention data reflecting changes in pain among FMS patients, including the mean and standard deviation (mean ± SD) of pain scores, as measured by instruments such as the Visual Analogue Scale (VAS). As noted earlier, outcome values were preferentially extracted from the most recent follow-up point immediately following the completion of the intervention.

### 2.4 Methodological quality assessment

The methodological quality of the included studies was assessed using two tools: the revised Cochrane Risk of Bias Tool for Randomized Trials (RoB2) and the Physiotherapy Evidence Database (PEDro) scale (Cashin and McAuley, 2020; Flemyng et al., 2023). The RoB2 tool evaluates seven domains of potential bias: random sequence generation (selection bias), allocation concealment (selection bias), blinding of participants and personnel (performance bias), blinding of outcome assessment (detection bias), incomplete outcome data (attrition bias), selective reporting (reporting bias), and other potential sources of bias (Sterne et al., 2019). The PEDro scale consists of 11 items, with studies scoring <4 points considered to be of poor quality, 4–5 points as fair, 6–8 points as good, and ≥9 points as excellent quality (Moseley et al., 2022).

### 2.5 Quality of the evidence

Two independent reviewers evaluated the strength of evidence for each outcome and subgroup using the Grading of Recommendations, Assessment, Development, and Evaluation (GRADE) system (Guyatt et al., 2008). The initial evidence was rated as high quality and downgraded based on the following criteria: i) publication bias, identified if there were at least 10 studies and a significant Egger test (p < 0.05) (Ioannidis and Trikalinos, 2007); ii) imprecision, when fewer than 400 participants were included in the meta-analysis (Mueller et al., 2007); iii) a high risk of bias in more than 25% of studies (PED score <6) (Higgins, 2019); iv) substantial heterogeneity (I^2^ > 50%) (Song, 1999). Confidence in the evidence was classified into four levels (very low, low, moderate, or high) based on the GRADE framework (Brożek et al., 2009).

### 2.6 Statistical analysis

#### 2.6.1 Meta-analysis

All statistical analyses were carried out using the “meta” package in R, while sensitivity analysis and publication bias assessment (via Egger’s test) were performed with Stata 15.0 (StataCorp, College Station). All statistical tests were two-tailed, with a significance threshold set at p < 0.05. The effect of AE on FMS pain was quantified by standardized mean differences (SMD) along with 95% confidence intervals (CI) (Bakbergenuly et al., 2020). When studies reported standard errors (SE) or 95% CIs instead of standard deviations (SD) (Takeshima et al., 2014), conversions were made according to the formulas provided in the Cochrane Handbook.

SD=SE ×n  and  SD=n×CIupper−CIlower3.92

Heterogeneity across studies was evaluated using the I^2^ statistic, where I^2^ values of less than 25% indicated minimal heterogeneity, 25%–50% represented low heterogeneity, 50%–75% indicated moderate heterogeneity, and I^2^ values exceeding 75% were considered high heterogeneity (Fletcher, 2022). A fixed-effect model was used when I^2^ was below 50%, and a random-effects model was applied when I^2^ was 50% or greater. If significant heterogeneity (I^2^ > 60%) was observed, subgroup and sensitivity analyses were performed to investigate potential sources of heterogeneity (Li and Reynolds, 1995).

#### 2.6.2 Subgroup analysis

To explore sources of heterogeneity in AE efficacy for fibromyalgia pain relief, we conducted pre-specified subgroup analyses stratified by seven clinically grounded moderators: (a) Agent group (Land-based aerobic exercises and Pool-based aerobic exercises), categorizing delivery settings; (b) Frequency dichotomized as two sessions/week and three sessions/week, based on ACSM recommendations; (c) Exercise session time tiered as short (<45 min), standard (45–60 min), and extended (≥60 min); (d) Exercise intensity classified by METs into low (<3 METs) and moderate (3–6 METs) domains per WHO activity strata; (e) Disease duration grouped as subacute (≤6 years) vs. chronic (>6 years) aligned with IASP pain chronification criteria; (f) Age segmented as young adults (18–45 years) and middle-aged (46–60 years) using UN population divisions; (g) Intervention duration: categorized into <12 weeks, 12–16 weeks, and ≥16 weeks. (h) Baseline pain severity stratified as moderate (<6/10) vs. severe (≥6/10) per IMMPACT cutoffs. These evidence-based stratifications, referenced from prior meta-analytic frameworks, aimed to identify effect modifiers for precision exercise prescription (Häuser et al., 2010; Tsai et al., 2024; Casanova-Rodríguez et al., 2025; Núñez-Cortés et al., 2025).

#### 2.6.3 Dose-response analysis

To quantify the dose-response relationship between exercise parameters and FMS pain reduction, we implemented model-based network meta-analysis (MBNMA) using the 'MBNMAdose’ package (v1.0.3) in R (Mawdsley et al., 2016). A Bayesian random-effects MBNMA approach was used to summarize associations between exercise intensity, duration, frequency, and their effects on FMS pain (Cleophas and Zwinderman, 2017). Given the Bayesian framework, all effect estimates were reported as posterior medians with corresponding 95% credible intervals (CrIs), which reflect the probability distributions of the model parameters. The consistency of the model and the connectivity of the network were assessed, and various nonlinear models were compared using fit indices, including the Deviance Information Criterion (DIC) (Wheeler et al., 2010). Nonlinear dose-response relationships were modeled using restricted cubic splines, as described in Supplementary Data 6.

## 3 Results

### 3.1 Study selection

**FIGURE 1:**
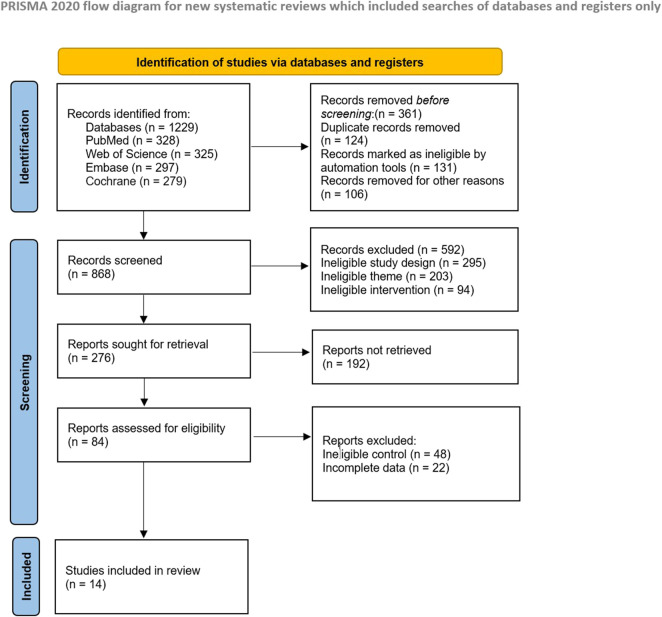
PRISMA flow diagram of the search process for studies.

The systematic search initially retrieved 1,229 records. After deduplication using Zotero 7.0 and manual verification, 868 unique records were screened by two independent reviewers based on abstracts. 84 full-text articles were evaluated for eligibility, with 14 randomized controlled trials (RCTs) meeting all inclusion criteria. The study selection process is detailed in the PRISMA flow diagram (Figure 1).

### 3.2 Study characteristics

The 14 included RCTs enrolled 777 fibromyalgia patients (99.4% female; pooled age: 48.4 ± 6.97 years; disease duration: 10.28 ± 6.16 years). Baseline pain severity (measured by 0–10 cm VAS) was balanced between AE (6.6 ± 1.7) and CON (6.5 ± 1.6). Exercise intensity was classified per ACSM METs thresholds: Light intensity (<3 METs; 2 RCTs), Moderate intensity (3–6 METs; 12 RCTs). Protocol specifications included: Duration: 4–32 weeks (median 12); Frequency: 2–3 sessions/week; Session time: 30–60 min (mean ± SD: 45.2 ± 8.7 min). Comprehensive study-level data are provided in Table 2.

**TABLE 2:** Characteristics of included studies.

Num	Study	Country	Clinical diagnosis	Agent	Sample size	Mean age	Female (%)	Pain	Symptom duration (years)	Intensity rating
1	Andrade et al. (2019)	Brazil	ACR 1990	AE	27 (27)	47 ± 8	100%	5.8 ± 2.7	6.5 ± 0.75	Moderate
				CON	27 (27)	48 ± 8	100%	5.4 ± 2.4	6.3 ± 0.83	
2	Baptista et al. (2012)	Spain	ACR 1990	AE	40 (39)	49.5	100%	7.7 ± 1.7	NA	Moderate
				CON	40 (39)	49.1	100%	7.5 ± 1.3	NA	
3	Hernando-Garijo et al. (2021)	Spain	ACR 1990	AE	17 (17)	51.81 ± 9.05	100%	7.08 ± 1.45	10.54 ± 7.4	Light
				CON	17 (17)	55.06 ± 8.51	100%	7.29 ± 1.07	10.54 ± 7.4	
4	Kayo et al. (2012)	USA	ACR 1990	AE	30 (30)	47.7 ± 5.3	100%	8.37 ± 1.45	12 ± 4	Light
				CON	30 (30)	46.1 ± 6.4	100%	8.62 ± 1.1	12 ± 10	
5	Letieri et al. (2013)	Brazil	ACR 1990	AE	30 (30)	58.2 ± 10.6	100%	6.1 ± 1.1	NA	Moderate
				CON	33 (33)	59.6 ± 9.4	100%	5.98 ± 1.3	NA	
6	Munguía-Izquierdo et al. (2007)	Spain	ACR 1990	AE	29 (29)	50 ± 7	100%	7.83 ± 1.85	14 ± 10	Moderate
				CON	24 (24)	46 ± 8	100%	7.25 ± 2.47	14 ± 9	
7	Sañudo et al. (2015)	Brazil	ACR 1990	AE	16 (16)	55 ± 2	100%	6.9 ± 1.8	NA	Moderate
				CON	12 (12)	58 ± 2	100%	7.6 ± 1.7	NA	
8	Tomas-Carus et al. (2008)	Portugal	ACR1990	AE	17 (15)	50.7 ± 10.6	100%	6.5 ± 1.9	20.1 ± 8.0	Moderate
				CON	16 (15)	50.9 ± 6.7	100%	6.4 ± 2.3	19.4 ± 6.9	
9	Tomas-Carus et al. (2007)	Spain	ACR 1990	AE	17 (17)	51 ± 10	100%	7.8 ± 1.6	14.7 ± 12.4	Moderate
				CON	17 (17)	51 ± 9	100%	7.1 ± 1.8	20.5 ± 13.3	
10	Van Eijk-Hustings et al. (2013)	Netherlands	ACR 1990	AE	47 (47)	43.9 ± 7.6	100%	6.2 ± 0.26	NA	Moderate
				CON	48 (48)	42.9 ± 11.0	100%	5.5 ± 0.2	NA	
11	Wigers et al. (1996)	Norway	ACR 1990	AE	20 (20)	42 ± 3.7	90%	7.2 ± 1.9	NA	Moderate
				CON	20 (20)	46 ± 2.1	95%	6.5 ± 1.7	NA	
12	Espí-López et al. (2016)	Spain	ACR 1990	AE	13 (9)	51.2 ± 5.5	92%	7.00 ± 1.68	NA	Moderate
				CON	13 (9)	57.1 ± 7.1	92%	6.33 ± 1.73	NA	
13	Rooks et al. (2007)	Israel	ACR 1990	AE	35 (35)	48 ± 11	100%	6.0 ± 2.1	5 ± 4	Moderate
				CON	38 (38)	50 ± 11	100%	6.0 ± 2.1	6 ± 5	
14	Schachter et al. (2003)	Canada	ACR 1990	AE	26 (26)	41.9 ± 8.57	100%	6.2 ± 2.54	3.5 ± 2.86	Moderate
				AE	29 (29)	41.3 ± 8.67	100%	5.7 ± 1.45	2.9 ± 2.76	Moderate
				CON	36 (36)	42.5 ± 6.69	100%	5.6 ± 2.02	3.6 ± 3.21	

ACR: american college of rheumatology; AE: aerobic exercise; CON: control; RCTs: Randomized controlled trials.

### 3.3 Meta-analysis results

**FIGURE 2:**
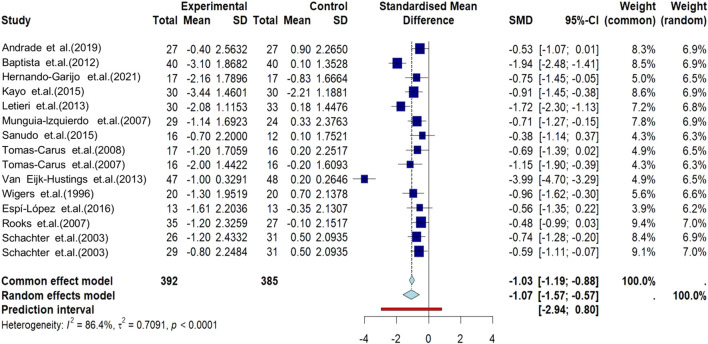
Meta-analysis of the effect of AE on pain in FMS participants. Forest plot illustrating the effect of AE on pain reduction in FMS participants compared to control groups.

Figure 2 forest plot of the effect of AE on pain reduction in fibromyalgia syndrome *versus* control groups. AE demonstrated significant pain reduction compared to controls [SMD: −1.07; 95% CI: −1.57 to −0.57; p < 0.0001] with substantial heterogeneity (I^2^ = 86.4%).

### 3.4 Subgroup analysis

**FIGURE 3:**
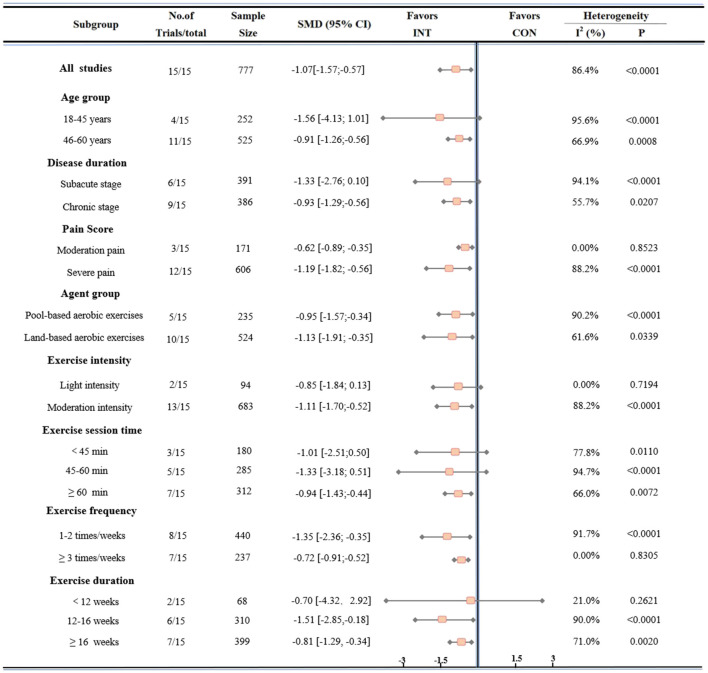
Subgroup analysis of the effect of AE on pain reduction in participants with FMS. The analysis was stratified by the following variables: age, disease duration, baseline pain score, medication use, exercise intensity, session duration, frequency, and overall intervention duration.

As shown in Figure 3, subgroup analyses indicated that both pool-based and land-based aerobic exercise were effective in reducing pain among patients with fibromyalgia syndrome (FMS). Pool-based aerobic exercise demonstrated a superior effect (SMD = −0.95; 95% CI: −1.57 to −0.34; p < 0.0001; I^2^ = 90.2%) compared to land-based aerobic exercise (SMD = −1.13; 95% CI: −1.91 to −0.35; p = 0.0339; I^2^ = 61.6%). Additionally, moderate-intensity aerobic exercise produced significant pain reduction (SMD = −1.11; 95% CI: −1.70 to −0.52; p < 0.0001; I^2^ = 88.2%), while low-intensity interventions did not achieve statistical significance (SMD = −0.85; 95% CI: −1.84 to 0.13; p = 0.7194; I^2^ = 0.0%). These findings suggest that moderate-intensity pool-based aerobic exercise is most effective for pain relief.

Further subgroup analyses based on participant characteristics revealed that younger patients (18–45 years) exhibited a greater effect size (SMD = −1.56; 95% CI: −4.13 to 1.01; p < 0.0001; I^2^ = 95.6%), although this result was not statistically significant. Middle-aged patients (46–60 years), however, experienced significant pain reduction (SMD = −0.91; 95% CI: −1.26 to −0.56; p = 0.0008; I^2^ = 66.9%). Regarding disease duration, individuals with a shorter disease history (≤6 years) showed a larger but not statistically significant effect (SMD = −1.33; 95% CI: −2.76 to 0.10; p < 0.0001; I^2^ = 94.1%), while those with longer disease duration (>6 years) achieved significant benefits (SMD = −0.93; 95% CI: −1.29 to −0.56; p = 0.0207; I^2^ = 55.7%). Moreover, participants with more severe baseline pain (≤6 points) demonstrated greater improvements (SMD = −1.19; 95% CI: −1.82 to −0.56; p < 0.0001; I^2^ = 88.2%) compared to those with moderate pain (>6 points) (SMD = −0.62; 95% CI: −0.89 to −0.35; p = 0.8523; I^2^ = 0.0%), although both groups benefited from aerobic exercise. In summary, aerobic exercise appears to be particularly effective for middle-aged FMS patients experiencing long-standing and severe pain.

Intervention parameters, including duration, frequency, and session time, also influenced treatment efficacy. Interventions lasting less than 12 weeks did not show significant pain reduction (SMD = −0.70; 95% CI: −4.32 to 2.92; p = 0.2621; I^2^ = 21.0%), while those lasting 12–16 weeks produced the most pronounced effects (SMD = −1.51; 95% CI: −2.85 to −0.18; p < 0.0001; I^2^ = 90.0%). Overall, interventions lasting 12 weeks or more remained significantly effective (SMD = −0.81; 95% CI: −1.29 to −0.34; p = 0.0020; I^2^ = 71.0%). Regarding exercise frequency, interventions performed two or fewer times per week were associated with greater pain reduction (SMD = −1.35; 95% CI: −2.36 to −0.35; p < 0.0001; I^2^ = 91.7%), while a frequency of three or more sessions per week was less effective (SMD = −0.72; 95% CI: −0.91 to −0.52; p = 0.8305; I^2^ = 0.0%). Session duration also played a role, with sessions lasting 60 min or longer yielding significant benefits (SMD = −0.94; 95% CI: −1.43 to −0.44; p = 0.0072; I^2^ = 66.0%), whereas shorter sessions (<60 min) did not reach statistical significance. In short, the most effective pain relief in FMS patients was achieved with interventions lasting 12–16 weeks, 1–2 times weekly, and 60 min per session.

### 3.5 Dose-response relationship

**FIGURE 4:**
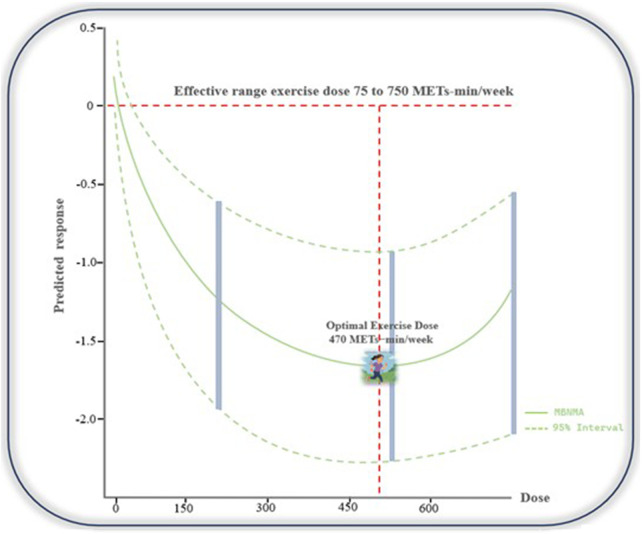
Dose–response relationship between AE volume and pain reduction in participants with FMS. The analysis indicates that AE doses ranging from 75 to 750 METs-min/week are effective in reducing pain, with an optimal dose identified at 470 METs-min/week.

The meta-analysis results indicate that the effectiveness of AE in alleviating pain among patients with FMS is influenced by the specific characteristics of the intervention protocol. To further explore this relationship, we conducted a dose–response analysis to evaluate the association between AE volume and pain reduction in FMS. As shown in Figure 4, the dose–response curve illustrates the relationship between AE dose and pain relief, with the green shaded area representing the distribution of sample sizes across different exercise doses. The findings suggest that AE doses ranging from 75 to 750 METs-min/week are effective in reducing pain in FMS patients. The optimal dose for pain relief was identified as 470 METs-min/week [SMD: −1.71 (95% CrI: −1.90 to −1.14)].

### 3.6 Sensitivity analysis

To assess the robustness of the findings, a leave-one-out sensitivity analysis was conducted. The results showed that excluding any single study did not substantially alter the overall effect size, which ranged from [SMD = −1.12 (95% CI: −1.56 to −0.67)] to [SMD = −0.88 (95% CI: −1.13 to −0.62)]. This indicates that the conclusions are stable and not driven by any individual study (Supplementary Data 5).

Notably, when two specific studies were removed, the heterogeneity decreased substantially (Baptista et al., 2012; Van Eijk-Hustings et al., 2013), yielding a pooled effect size of [SMD = −0.80 (95% CI: −0.99 to −0.60)], with I^2^ = 24.4% (p = 0.197). This suggests that these studies contributed significantly to the observed heterogeneity. However, the direction and statistical significance of the pool.

### 3.7 Meta regression

To explore potential sources of heterogeneity in effect sizes, meta-regression analyses were performed using multiple covariates (Van Houwelingen et al., 2002). The results indicated that none of the examined variables significantly accounted for the between-study heterogeneity: disease type (p = 0.9209), baseline pain score (p = 0.9209), intervention duration (p = 0.7714), intervention frequency (p = 0.1518), session duration (p = 0.7134), intervention intensity score (p = 0.6806), and intervention modality (p = 0.7225). These findings suggest that the observed heterogeneity may be attributable to other unmeasured factors not captured in the current analysis. Meta regression results are in Supplementary Data 4.

### 3.8 Risk of bias

Using the Cochrane RoB 2.0 tool, methodological quality assessment revealed: Low risk: nine studies (64.3%); Some concerns: three studies (21.4%); High risk: two studies (14.3%). A comprehensive risk-of-bias heatmap visualizing domain-specific judgments across all 14 studies is provided in Supplementary Data 8.

The methodological quality was additionally assessed using the 11-point PEDro scale. Results indicated: High quality (score ≥9/11): 3 RCTs (21.4%); Good quality (score 6–8/11): 11 RCTs (78.6%). Domain-specific scores and quality thresholds are detailed in Supplementary Data 9.

### 3.9 Publication bias

**FIGURE 5:**
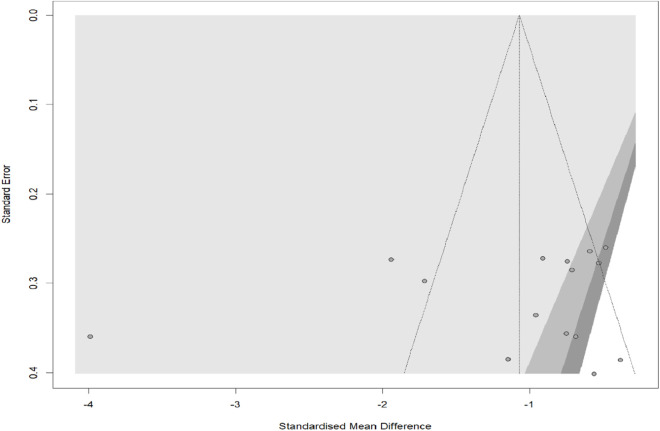
Funnel plot assessing publication bias in the included studies. Visual inspection of the funnel plot revealed no noticeable asymmetry, suggesting a minimal risk of publication bias.

Publication bias was evaluated using a funnel plot, as shown in Figure 5. Upon visual inspection, the funnel plot showed no noticeable asymmetry, suggesting a minimal risk of publication bias. Moreover, Egger’s test revealed no significant small-study effects (p = 0.531), indicating that studies with smaller sample sizes had little influence on the overall findings.

## 4 Discussion

### 4.1 Main findings

This meta-analysis included 14 studies with a total of 777 participants aged between 41 and 60, aiming to evaluate the effect of AE on pain in patients with FMS. Our results showed that AE significantly alleviated pain after the intervention, highlighting its effectiveness in relieving FMS-related pain. In addition, subgroup analysis revealed that moderate-intensity pool-based aerobic exercise, performed 1–2 times weekly for ≥60 min per session throughout 12–16 weeks, significantly improved chronic, severe pain in middle-aged FMS patients. Finally, and most importantly, we explored the dose-response relationship of AE in alleviating FMS pain. Using METs for dose-response analysis, we identified an effective dose range of 75–750 METs-min/week, with an optimal dose of 470 METs-min/week, and 750 METs-min/week still showing clinical significance.

Despite the significant benefits of AE in alleviating pain in patients with FMS, the meta-analysis results still exhibit considerable statistical heterogeneity (I^2^ = 86.4%). Such a level of heterogeneity is not uncommon in complex behavioral intervention studies conducted across multiple countries and clinical settings (Meyer et al., 2007). Variations in participant characteristics (such as age and baseline symptom severity), intervention methods (such as exercise modality, level of supervision, and implementation environment), and outcome measures (such as pain type and assessment tools) may contribute to the variability in the results (Song, 1999). To explore the potential sources of heterogeneity, we conducted a series of pre-planned subgroup analyses examining moderating variables such as agent group, age, disease duration, baseline pain score, and exercise intervention parameters. Several key factors that may explain part of the variability were identified (Thompson, 1994). For instance, the studies included categorized the exercise modalities into pool-based aerobic exercises and land-based aerobic exercises. These two modalities differ significantly in terms of physiological load, social interaction, and environmental adaptability, which may further influence intervention outcomes and increase variability between studies (Churchland et al., 2006). However, due to the limited number of studies for each specific exercise modality, we were only able to perform subgroup analyses for some exercise forms. Future research should focus on using standardized intervention reporting frameworks to systematically assess the independent efficacy of different types of AE, improving the comparability of studies and their clinical relevance (Häuser et al., 2010). Additionally, sensitivity analyses, excluding outliers, still yielded consistent results, further enhancing the robustness of our conclusions. In conclusion, although high heterogeneity suggests caution when interpreting the results, it also emphasizes the necessity and practical significance of tailoring exercise interventions based on patient characteristics and background context in real-world clinical applications.

The mechanisms by which AE alleviates pain in patients with FMS can be explored from several perspectives. First, the activation of exercise-induced hypoalgesia (Sluka et al., 2018). Moderate-intensity and sustained AE can stimulate the central nervous system to release endogenous opioids such as endorphins, thereby increasing pain thresholds, inhibiting central sensitization, and relieving chronic pain. Previous studies have confirmed that the exercise-induced hypoalgesia mechanism remains effectively responsive in individuals with FMS (Martins-Pinge, 2011; Schoenfeld and Swanson, 2021). Second, modulation of neurotransmitter activity and neural regulation (Heijnen et al., 2016). FMS is often associated with dysfunction of central neurotransmitters, which negatively affects pain regulation and emotional stability (Berwick et al., 2022). AE has been shown to upregulate dopaminergic activity and enhance the reuptake and modulation of various neurotransmitters, thereby contributing to pain relief from a neurochemical perspective while also improving emotional wellbeing and reducing psychological stress (Weicker and Strüder, 2001; Evancho et al., 2023). In addition, improvement of muscle metabolism and circulation to alleviate myogenic pain (Søgaard and Sjøgaard, 2017). Previous RCTs have demonstrated that regular AE in FMS patients can enhance muscle oxidative capacity, improve local blood flow, reduce muscle soreness and stiffness, and increase tolerance to daily physical activity, ultimately decreasing the frequency and severity of pain episodes (Dobson et al., 2014; King and Estill, 2019).

Subgroup analyses provided valuable insights into how age, disease duration, and baseline pain status affect intervention outcomes. Compared to younger patients, AE was significantly more effective in alleviating long-term severe pain in middle-aged FMS patients, which is consistent with previous studies, indicating that the benefits of exercise in pain relief are more pronounced in older individuals and those with long-term severe pain (Thomas and Blotman, 2010). This difference may be attributed to the fact that when middle-aged FMS patients are in a decompensated borderline state, the physiological remodeling, neuroregulation, and anti-inflammatory effects triggered by exercise can maximize the leverage effect, thus resulting in significant pain relief (Busch et al., 2007). In contrast, for those with milder pain, the impact of these physiological changes may be less pronounced, leading to more modest improvements. This further underscores the importance of tailoring exercise interventions based on the severity of pain symptoms.

Additionally, the type of intervention used differed in its effectiveness in relieving FMS pain. Our subgroup analysis indicated that, compared to land-based aerobic exercise, pool-based aerobic exercise provided more effective pain relief for FMS patients, as confirmed by previous studies (Galvão-Moreira et al., 2021). This is due to the unique biomechanical and neurophysiological regulation of the water environment. Buoyancy in water unloads 90% of the joint load, reducing the impact of exercise on pain. The thermal effect directly inhibits pain transmission and improves muscle microcirculation, thereby activating the release of endogenous analgesics and anti-inflammatory effects, creating a beneficial pain-free activation cycle (Acosta-Gallego, 2018).

Furthermore, differences in exercise programs play a crucial role in relieving pain in FMS patients. While earlier studies generally suggested that moderate-intensity programs of 2–3 sessions per week can reduce pain symptoms (Häuser et al., 2010; Casanova-Rodríguez et al., 2025), our findings refine and, in some respects, diverge from these recommendations. Specifically, we observed that moderate-intensity AE performed only 1–2 times weekly, provided each session lasted at least 60 min over a 12–16 weeks period, was sufficient to achieve clinically meaningful pain reduction. This contrasts with the higher frequencies previously emphasized, and suggests that adequate weekly exercise volume, rather than frequency *per se*, is the critical determinant of therapeutic benefit. Such insights highlight the importance of tailoring exercise prescriptions to patient tolerance, minimizing the risk of overtraining and fatigue while still ensuring sufficient stimulus for pain relief.

The dose-response analysis revealed significant effects, emphasizing the importance of determining the optimal exercise dose. The findings suggest that the effective dose range for reducing FMS pain is between 75 and 750 METs-min/week, with approximately 470 METs-min/week being the most effective dose. As shown in Figure 4, therapeutic benefits are maintained even at 750 METs-min/week. This may be due to higher doses of AE enhancing the body’s anti-inflammatory responses, boosting the endogenous analgesic system, and modulating central sensitization, which collectively contribute to pain relief (Dobson et al., 2014; Sluka et al., 2018; Schoenfeld and Swanson, 2021). However, exceeding this range may reduce the benefits or lead to adverse effects from overtraining or fatigue (Thomas and Blotman, 2010). These findings are crucial for designing exercise interventions, highlighting the need for individualized exercise programs that consider both intensity and volume to optimize outcomes. Personalized exercise prescriptions can help mitigate the risks of overtraining or undertraining (Lucini et al., 2024). Future studies should explore how individual factors, such as baseline health and pain status, influence the dose-response relationship to enhance the effectiveness of exercise-based health interventions.

### 4.2 Strengths

First, this study pioneers the application of an AE-only inclusion criterion, rigorously excluding confounding interventions such as resistance training, stretching, and mind-body exercises, thereby precisely quantifying the independent effect of AE on pain in FMS. This design effectively addresses the issue of effect size contamination caused by mixed interventions in previous meta-analyses by Häuser et al. (2010) and Casanova-Rodríguez et al. (2025) (Häuser et al., 2010; Núñez-Cortés et al., 2025).

This study is the first to quantitatively establish a dose-response relationship between AE and pain reduction in FMS by combining exercise intensity and duration through METs. This model overcomes the limitations of Núñez-Cortés et al. (2025), which relied solely on exercise duration to define dosage, thereby providing precise evidence-based guidance for developing individualized exercise prescriptions in clinical practice (Casanova-Rodríguez et al., 2025).

Furthermore, the study comprehensively explored the key regulatory factors such as age, baseline pain score, and course of disease. Combined with the intervention characteristics of exercise type, cycle, frequency, and intensity, a multi-dimensional analysis framework was constructed to reveal the applicable conditions of the best exercise program.

Finally, the effects were assessed based on RCT data, avoiding the risk of placebo effects inherent in pre-post comparisons. The resulting dose-response relationship can be directly translated into a stepwise exercise prescription, advancing the precision-based clinical management of FMS.

### 4.3 Limitations

Firstly, although the study is restricted to AE only, the specific exercise modalities were categorized into land-based and pool-based aerobic exercise. The biomechanical characteristics of these different modalities may impact pain modulation pathways. Future research should further subdivide exercise types and conduct subgroup analyses.

Secondly, while the study offers a dose-response relationship for AE in pain relief, some of the reported exercise doses may lack precision, particularly when relying on self-reported data. This could introduce uncertainty in the dose-response model estimates, potentially compromising the accuracy of the determination of the optimal dose.

Finally, many of the included studies had short follow-up periods, which did not allow for a comprehensive assessment of the long-term effects of exercise interventions on fibromyalgia patients. Furthermore, differences in follow-up durations across studies may impact the stability of the meta-analysis results.

## 5 Conclusion

This meta-analysis demonstrates that AE significantly reduces pain symptoms in individuals with FMS. The effect is particularly evident among patients aged 45–60 and those with clinically confirmed chronic severe pain. Moderate-intensity, pool-based aerobic exercise (3.0–6.0 METs) appears most effective when prescribed for 60 min per session, 1–2 times per week, over a 12–16 weeks period. Dose-response analysis indicated an effective range of 75–750 METs-min/week, with an optimal dose of approximately 470 METs-min/week. These results offer valuable guidance for developing tailored exercise interventions aimed at pain management in this population.

## Data Availability

The original contributions presented in the study are included in the article/Supplementary Material, further inquiries can be directed to the corresponding authors.
