# Supplementary material for: Optimal aerobic exercise dose for pain relief in fibromyalgia syndrome: a systematic review and meta-analysis
Source: Front Physiol. 2025 Dec 8;16:1693735. doi: 10.3389/fphys.2025.1693735 (PMC12719272; doi:10.3389/fphys.2025.1693735)
Supplement: Supplementary file 1 [file DataSheet1.docx]

Supplementary Material

[1 Supplementary Data: Search Strategy 2](#_Toc205803681)

[2 Supplementary Data: Characteristics of Included Studies 4](#_Toc205803682)

[3 Supplementary Data: Characteristics of Studies and Subject Included Study of the Dataset 9](#_Toc205803683)

[4 Supplementary Data: Meta regression 10](#_Toc205803684)

[5 Supplementary Data: Sensitivity Analysis Result 14](#_Toc205803685)

[6 Supplementary Data: Network Meta Dose-Response Analysis 14](#_Toc205803686)

[7 Supplementary Data: Non-Linear Functions and Models Fit Comparison 18](#_Toc205803690)

[8 Supplementary Data: Risk of Bias of Rob2 Assessment 22](#_Toc205803691)

[9 Supplementary Data: PEDro Scale 24](#_Toc205803692)

# Supplementary Data: Search Strategy

**Table 1.1** PubMed Search Strategy.

| **Search Terms** | \|  \| \| --- \|  \| **Results** \| \| --- \| |
| --- | --- | --- | --- |
| ("fibromyalgia"[Mesh] OR fibromyalgia[tiab] OR "fibromyalgia syndrome"[tiab]) | 15717 |
| AND ("aerobic exercise"[Mesh] OR "aerobic training"[tiab] OR "aerobic exercise"[tiab] OR "endurance training"[tiab]) | 873 |
| AND ("pain"[Mesh] OR pain[tiab] OR "pain relief"[tiab] OR "pain management"[tiab]) | 443 |
| AND (randomized controlled trial[pt] OR controlled clinical trial[pt] OR randomized[tiab] OR placebo[tiab]) | 328 |

**Table 1.2** Embase Search Strategy.

| **Search Terms** | \|  \| \| --- \|  \| **Results** \| \| --- \| |
| --- | --- | --- | --- |
| ('fibromyalgia'/exp OR fibromyalgia:ti,ab OR 'fibromyalgia syndrome':ti,ab) | 13462 |
| AND ('aerobic exercise'/exp OR 'aerobic training':ti,ab OR 'endurance training':ti,ab) | 886 |
| AND ('pain'/exp OR pain:ti,ab OR 'pain relief':ti,ab) | 584 |
| AND ('randomized controlled trial'/exp OR 'randomized':ti ': ti,ab OR 'placebo':ti,ab) | 297 |

**Table 1.3** Web of Science Search Strategy.

| **Search Terms** | \|  \| \| --- \|  \| **Results** \| \| --- \| |
| --- | --- | --- | --- |
| TS=(fibromyalgia OR "fibromyalgia syndrome") | 18470 |
| AND TS=("aerobic exercise" OR "aerobic training" OR "endurance training") | 994 |
| AND TS=(pain OR "pain relief" OR "pain management") | 669 |
| AND TS=("randomized controlled trial" OR randomized OR placebo) | 325 |

**Table 1.4** Cochrane Library Search Strategy.

| **Search Terms** | \|  \| \| --- \|  \| **Results** \| \| --- \| |
| --- | --- | --- | --- |
| [fibromyalgia]:ti,ab,kw | 9564 |
| AND [aerobic exercise OR aerobic training OR endurance training]:ti,ab,kw | 651 |
| AND [pain OR pain relief OR pain management]:ti,ab,kw | 279 |

# Supplementary Data: Characteristics of Included Studies

| **Num** | **Study** | **Country** | **Clinical Diagnosis** | **Agent** | **Sample size** | **Mean age** | **Female（%）** | **Pain** | **Symptom duration(years)** | **Intervention Detail** | **Intensity** | | **Outcomes** |
| --- | --- | --- | --- | --- | --- | --- | --- | --- | --- | --- | --- | --- | --- |
|  |  |  |  |  |  |  |  |  |  |  | **Intensity contents** | **Intensity rating** |  |
| **1** | Andrade et al. (2019) | Brazil | ACR 1990 | AE | 27(27) | 47 ± 8 | 100% | 5.8 ± 2.7 | 6.5 ± 0.75 | Aquatic aerobics exercises. | 4 W power x 4 min, gradually increasing the peak motion, and stopping when it cannot be maintained at 60 rpm. | Moderate | Pain |
|  |  |  |  | CON | 27(27) | 48 ± 8 | 100% | 5.4 ± 2.4 | 6.3 ± 0.83 |  |  |  |  |
| **2** | Baptista et.al. (2012) | Spain | ACR 1990 | AE | 40(39) | 49.5 | 100% | 7.7 ± 1.7 | NA | Aerobic includes: dance and choreography; relaxation | Not report | Moderate | Pain |
|  |  |  |  | CON | 40(39) | 49.1 | 100% | 7.5 ± 1.3 | NA |  |  |  |  |
| **3** | Hernando-Garijo et.al. (2021) | Spain | ACR 2016 | AE | 17(17) | 51.81 ± 9.05 | 100% | 7.08 ± 1.45 | 10.54 ± 7.4 | Aerobic exercise includes: active stretching; low-intensity rhythmic aerobic exercise | Intensity increases if the Borg scale is less than 4 and decreases if the Borg scale is greater than 7. | Light | Pain |
|  |  |  |  | CON | 17(17) | 55.06 ± 8.51 | 100% | 7.29 ± 1.07 | 10.54 ± 7.4 |  |  |  |  |
| **4** | Kayo et.al. (2015) | USA | ACR 1990 | AE | 30(30) | 47.7 ± 5.3 | 100% | 8.37 ± 1.45 | 12 ± 4 | Aerobic exercises include: walking, training | Every 4 weeks, the duration of the walk increases (from 25-30 minutes to 50 minutes), as does the intensity (starting at 40-50% of heart rate), and at week 16 (60-70% of heart rate). | Light | Pain |
|  |  |  |  | CON | 30(30) | 46.1 ± 6.4 | 100% | 8.62 ± 1.1 | 12 ± 10 |  |  |  |  |
| **5** | Letieri et.al. (2013) | Brazil | ACR 1990 | AE | 30(30) | 58.2 ± 10.6 | 100% | 6.1 ± 1.1 | NA | Aquatic aerobics exercises. | Exercise intensity was adjusted by Pse, with targets set at Pse of 6 and 7, which needed to be maintained at 60-70% of maximum heart rate | Moderate | Pain |
|  |  |  |  | CON | 33(33) | 59.6 ± 9.4 | 100% | 5.98 ± 1.3 | NA |  |  |  |  |
| **6** | Munguía-Izquierdo et.al. (2007) | Spain | ACR 1990 | AE | 29(29) | 50 ± 7 | 100% | 7.83 ± 1.85 | 14 ± 10 | Based on the aquatic environment, including aerobic exercise | Aerobic:1-2 weeks 50%-60% of maximum heart rate;2-4 weeks 55%-65% of maximum heart rate;5-8 weeks 60%-70% of maximum heart rate;9-12 weeks 65%-75% of maximum heart rate;13-16 weeks 70%-80% of maximum heart rate. | Moderate | Pain |
|  |  |  |  | CON | 24(24) | 46 ± 8 | 100% | 7.25 ± 2.47 | 14 ± 9 |  |  |  |  |
| **7** | Sañudo et.al. (2015) | Brazil | ACR 1990 | AE | 16(16) | 55 ± 2 | 100% | 6.9 ± 1.8 | NA | Aerobic exercises include walking and jogging | Aerobic exercise, 15-20 minutes of steady-state exercise at 60-65% of predicted maximal heart rate and 15 minutes of interval training at 75-80% (six 1.5-minute repetitions with rest intervals inserted within 1 minute) | Moderate | Pain |
|  |  |  |  | CON | 12(12) | 58 ± 2 | 100% | 7.6 ± 1.7 | NA |  |  |  |  |
| **8** | Tomas-Carus et.al. (2008) | Portugal | ACR1990 | AE | 17(15) | 50.7 ± 10.6 | 100% | 6.5 ± 1.9 | 20.1 ± 8.0 | Aquatic environment-based exercises include step aerobics. | Aquatic exercise consists of 10 minutes of aerobic exercise, Stretch at 60-65% of maximum heart rate. | Moderate | Pain |
|  |  |  |  | CON | 16(15) | 50.9 ± 6.7 | 100% | 6.4 ± 2.3 | 19.4 ± 6.9 |  |  |  |  |
| **9** | Tomas-Carus et.al. (2007) | Spain | ACR 1990 | AE | 17(17) | 51 ± 10 | 100% | 7.8 ± 1.6 | 14.7 ± 12.4 | Exercises based on the aquatic environment include aerobic. | Aquatic exercise: 10 minutes of aerobic exercise at 65-75% of maximum heart rate. | Moderate | Pain |
|  |  |  |  | CON | 17(17) | 51 ± 9 | 100% | 7.1 ± 1.8 | 20.5 ± 13.3 |  |  |  |  |
| **10** | Van Eijk-Hustings et.al. (2013) | Netherlands | ACR 1990 | AE | 47(47) | 43.9 ± 7.6 | 100% | 6.2 ± 0.26 | NA | Aerobic exercises include: walking, jogging | The low-intensity aerobic portion was designed to achieve 55-64% of predicted maximum heart rate. | Moderate | Pain |
|  |  |  |  | CON | 48(48) | 42.9 ± 11.0 | 100% | 5.5 ± 0.2 | NA |  |  |  |  |
| **11** | Wigers et.al. (1996) | Norway | ACR 1990 | AE | 20(20) | 42 ± 3.7 | 90% | 7.2 ± 1.9 | NA | Aerobic exercises include: walking, aerobic games. | At 60-70% of maximum heart rate, the cadence was gradually increased to four stages of high-intensity training and then decreased from four stages. | Moderate | Pain |
|  |  |  |  | CON | 20(20) | 46 ± 2.1 | 95% | 6.5 ± 1.7 | NA |  |  |  |  |
| **12** | Espí-Lópezet .al.(2016) | Spain | ACR 2010 | AE | 13(9) | 51.2 ± 5.5 | 92% | 7.00 ± 1.68 | NA | Aerobic includes: low-impact exercise | Low-impact cardio is dynamic and fluid (30 minutes), Low intensity | Moderate | Pain |
|  |  |  |  | CON | 13(9) | 57.1 ± 7.1 | 92% | 6.33 ± 1.73 | NA |  |  |  |  |
| **13** | Rooks et.al. (2007) | Israel | ACR 1990 | AE | 35(35) | 48 ± 11 | 100% | 6.0 ± 2.1 | 5 ± 4 | Gradual walk | Not report | Moderate | Pain |
|  |  |  |  | CON | 38(38) | 50 ± 11 | 100% | 6.0 ± 2.1 | 6 ± 5 |  |  |  |  |
| **14** | Schachter et.al. (2003) | Canada | ACR 1990 | AE | 26(26) | 41.9 ± 8.57 | 100% | 6.2 ± 2.54 | 3.5 ± 2.86 | Long bout of aerobic exercise. | Target exercise intensity began at 40 % to 50 % of HRR in the first week, progressed to 65 % to 75 % of HRR in the 12th week, and remained at this level from the 12th week to the 16th week. | Moderate | Pain |
|  |  |  |  | AE | 29(29) | 41.3 ± 8.67 | 100% | 5.7 ± 1.45 | 2.9 ± 2.76 | A short bout of aerobic exercise | Target exercise intensity began at 40 % to 50 % of HR in the first week, progressed to 65 % to 75 % of HR in the 12th week, and remained at this level from the 12th week to the 16th week. | Moderate |  |
|  |  |  |  | CON | 36(36) | 42.5 ± 6.69 | 100% | 5.6 ± 2.02 | 3.6 ± 3.21 |  |  |  |  |

# Supplementary Data: Characteristics of Studies and Subject Included Study of the Dataset

We calculated the weekly METs-min $(METs*min/week = duration in minutes \times times per week \times MET value)$, which considers not only the duration and frequency of exercise but also the intensity.

| **Id** | **y** | **SE** | **SD** | **n** | **Stimated dose** | **Residual dose** | **Mets-min/week** | **METs** | **Code** | **Total MET** | **Duration (Weeks)** | **Frequency (x/Week)** | **Min** |
| --- | --- | --- | --- | --- | --- | --- | --- | --- | --- | --- | --- | --- | --- |
| 1 | -0.4 | 0.493288 | 2.563 | 27 | 250 | 92 | 342 | 3.8 | 18356 | 5472 | 16 | 2 | 45 |
| 1 | 0.9 | 0.43589 | 2.265 | 27 | 0 | 0 | 0 |  |  | 0 |  |  |  |
| 2 | -3.1 | 0.295381 | 1.868 | 40 | 500 | 40 | 540 | 4.5 | 03025 | 8640 | 16 | 2 | 60 |
| 2 | 0.1 | 0.213892 | 1.353 | 40 | 0 | 0 | 0 |  |  | 0 |  |  |  |
| 3 | -2.16 | 0.43403 | 1.790 | 17 | 250 | 0 | 250 | 2.5 | 02140 | 3750 | 15 | 2 | 50 |
| 3 | -0.83 | 0.404162 | 1.666 | 17 | 0 | 0 | 0 |  |  | 0 |  |  |  |
| 4 | -3.44 | 0.266577 | 1.460 | 30 | 250 | 26 | 276 | 2.3 | 17150 | 3312 | 12 | 2 | 60 |
| 4 | -2.21 | 0.216918 | 1.188 | 30 | 0 | 0 | 0 |  |  | 0 |  |  |  |
| 5 | -2.08 | 0.203625 | 1.115 | 30 | 250 | -22 | 228 | 3.8 | 18356 | 3420 | 15 | 2 | 30 |
| 5 | 0.18 | 0.251998 | 1.448 | 33 | 0 | 0 | 0 |  |  | 0 |  |  |  |
| 6 | -1.14 | 0.314253 | 1.692 | 29 | 750 | -66 | 684 | 3.8 | 18356 | 10944 | 16 | 3 | 60 |
| 6 | 0.33 | 0.485064 | 2.376 | 24 | 0 | 0 | 0 |  |  | 0 |  |  |  |
| 7 | -0.7 | 0.55 | 2.200 | 16 | 500 | -80 | 420 | 3.5 | 02034 | 1680 | 4 | 2 | 60 |
| 7 | 0.1 | 0.5058 | 1.752 | 12 | 0 | 0 | 0 |  |  | 0 |  |  |  |
| 8 | -1.2 | 0.413735 | 1.706 | 17 | 750 | -66 | 684 | 3.8 | 18356 | 21888 | 32 | 3 | 60 |
| 8 | 0.2 | 0.562917 | 2.252 | 16 | 0 | 0 | 0 |  |  | 0 |  |  |  |
| 9 | -2 | 0.360555 | 1.442 | 16 | 750 | -66 | 684 | 3.8 | 18356 | 8208 | 12 | 3 | 60 |
| 9 | -0.2 | 0.402337 | 1.609 | 16 | 0 | 0 | 0 |  |  | 0 |  |  |  |
| 10 | -1 | 0.048003 | 0.329 | 47 | 500 | -68 | 432 | 4.8 | 02005 | 5184 | 12 | 2 | 45 |
| 10 | 0.2 | 0.038188 | 0.265 | 48 | 0 | 0 | 0 |  |  | 0 |  |  |  |
| 11 | -1.3 | 0.436463 | 1.952 | 20 | 750 | -102 | 648 | 4.8 | 02005 | 9072 | 14 | 3 | 45 |
| 11 | 0.7 | 0.478017 | 2.138 | 20 | 0 | 0 | 0 |  |  | 0 |  |  |  |
| 12 | -1.61 | 0.611172 | 2.204 | 13 | 500 | 76 | 576 | 4.8 | 02005 | 4608 | 8 | 2 | 60 |
| 12 | -0.35 | 0.590951 | 2.131 | 13 | 0 | 0 | 0 |  |  | 0 |  |  |  |
| 13 | -1.2 | 0.393156 | 2.325 | 35 | 750 | 60 | 810 | 6 | 12010 | 12960 | 16 | 3 | 45 |
| 13 | -0.1 | 0.414103 | 2.151 | 27 | 0 | 0 | 0 |  |  | 0 |  |  |  |
| 14 | -1.2 | 0.477183 | 2.433 | 26 | 500 | -68 | 432 | 4.8 | 02005 | 6912 | 16 | 3 | 30 |
| 14 | 0.5 | 0.376006 | 2.093 | 31 | 0 | 0 | 0 |  |  | 0 |  |  |  |
| 15 | -0.8 | 0.417509 | 2.248 | 29 | 500 | -284 | 216 | 4.8 | 02005 | 3456 | 16 | 2 | 15 |
| 15 | 0.5 | 0.376006 | 2.093 | 31 | 0 | 0 | 0 |  |  | 0 |  |  |  |

**Note: Agent:** Aerobic exercise; **Se:** Standard error; **y:** mean change indicates the mean change from the baseline of the evaluation tool; **n:** Sample size; **SD:** Standard deviation**; Exact dose:** the group of doses by approximation; **Mets-min/week:** the exact estimated METs per week that participants accumulated in the study residual; **Total MET:** the account of met in follow-up period; **Period:** the account exercise of weeks in different study; **Frequency:** the account exercise of days those participants; **Min:** how many minutes a session lasted; **Met:** metabolic equivalent of task.

# Supplementary Data: Meta regression


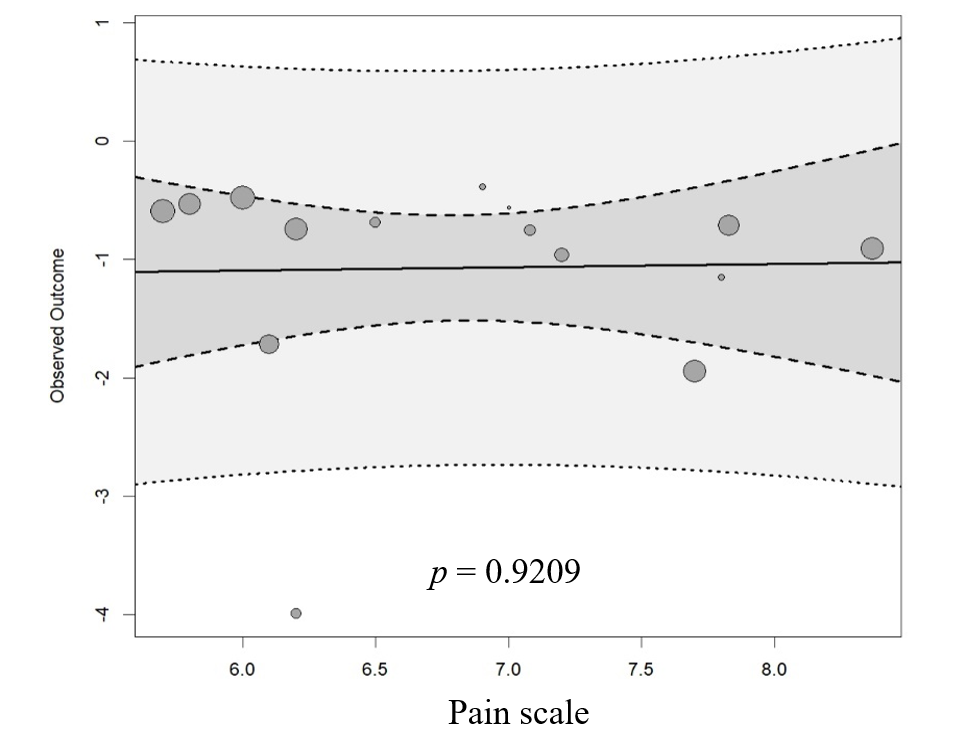


**Figure 4.1 Meta-regression analysis result of the Pain scale.**


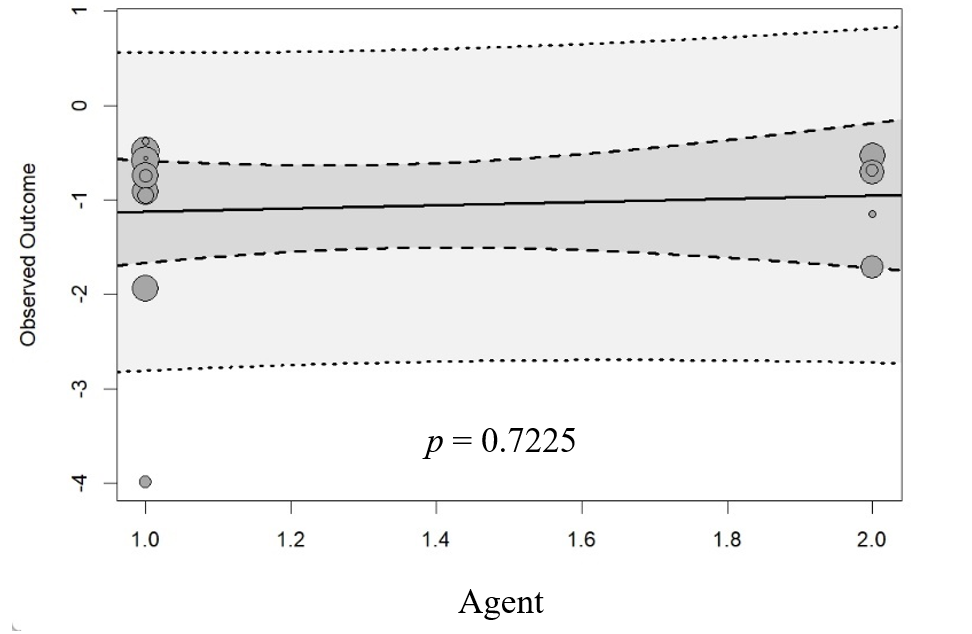


**Figure 4.2 Meta-regression analysis result of Agent.**


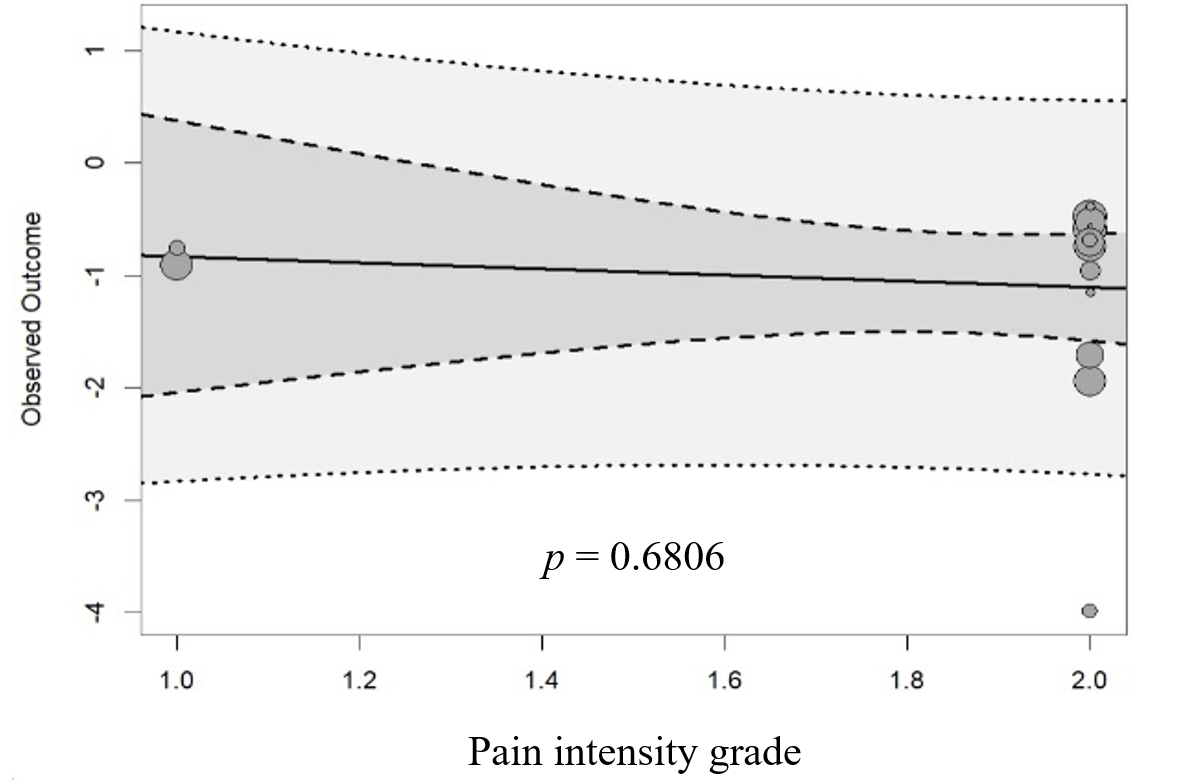


**Figure 4.3 Meta-regression analysis result of Pain intensity grade.**


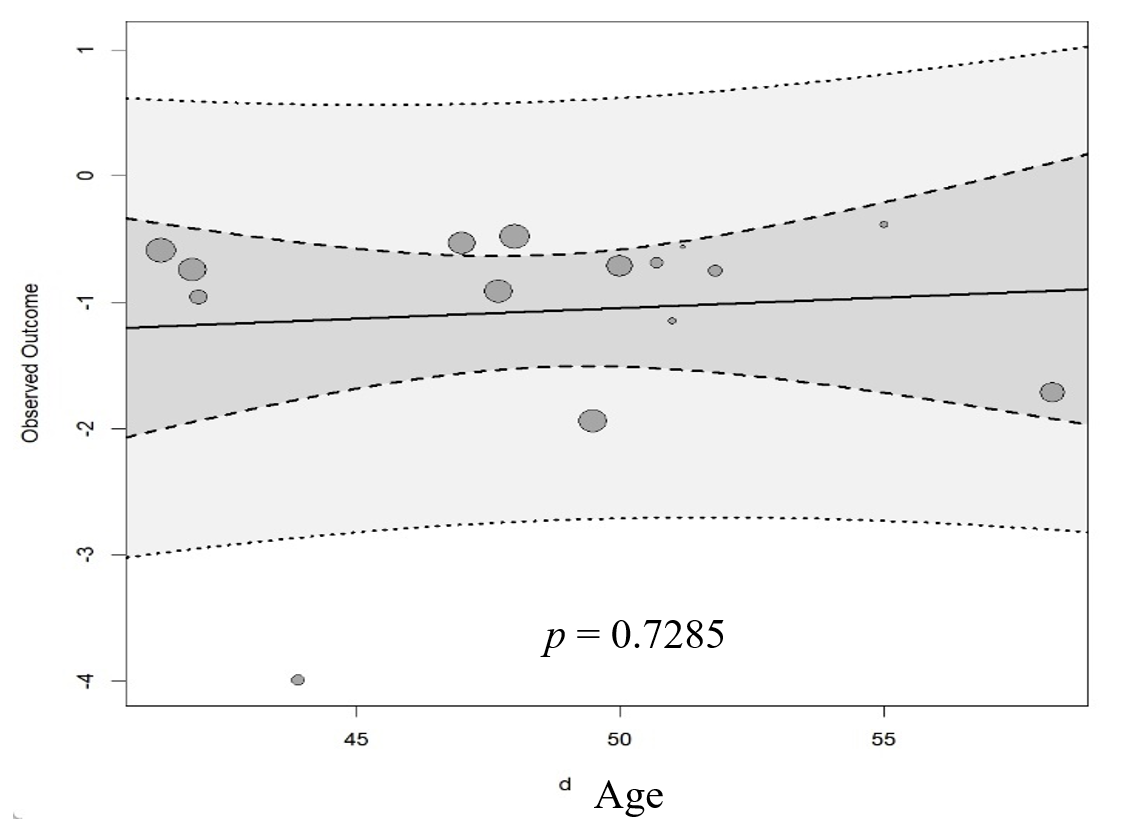


**Figure 4.4 Meta-regression analysis result of Age.**


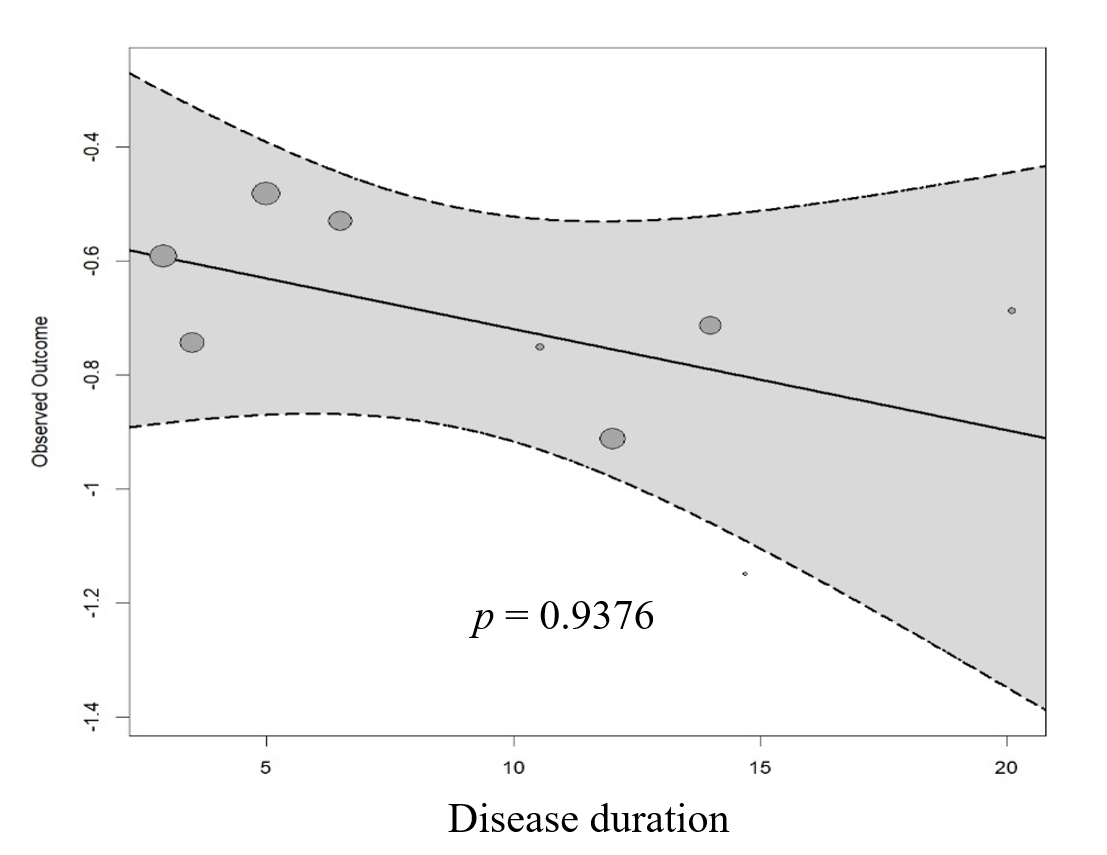


**Figure 4.5 Meta-regression analysis result of Disease duration.**
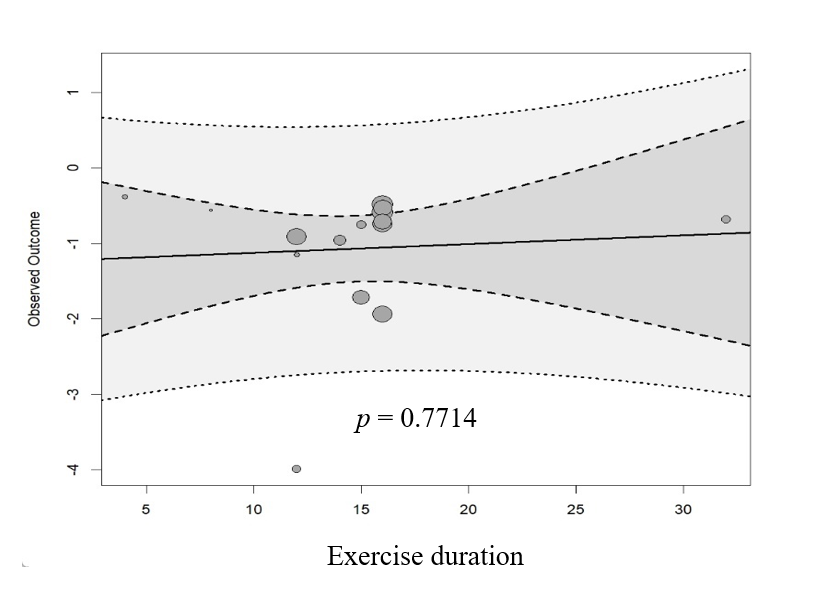


**Figure 4.6 Meta-regression analysis result of Exercise Duration.**


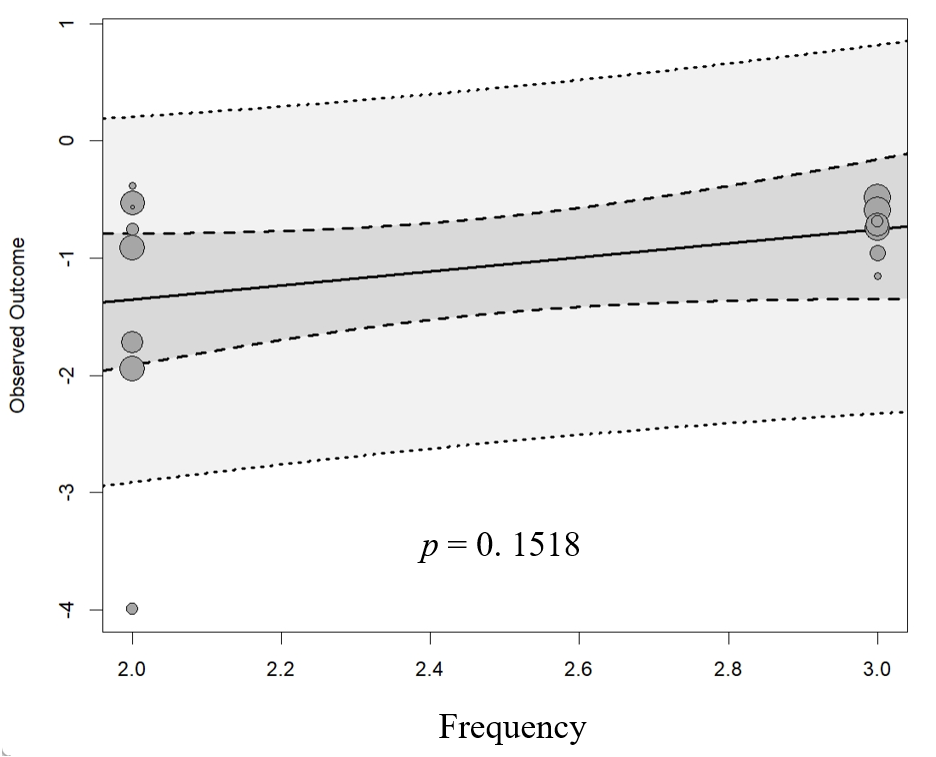
**Figure 4.7 Meta-regression analysis result of Frequency.**


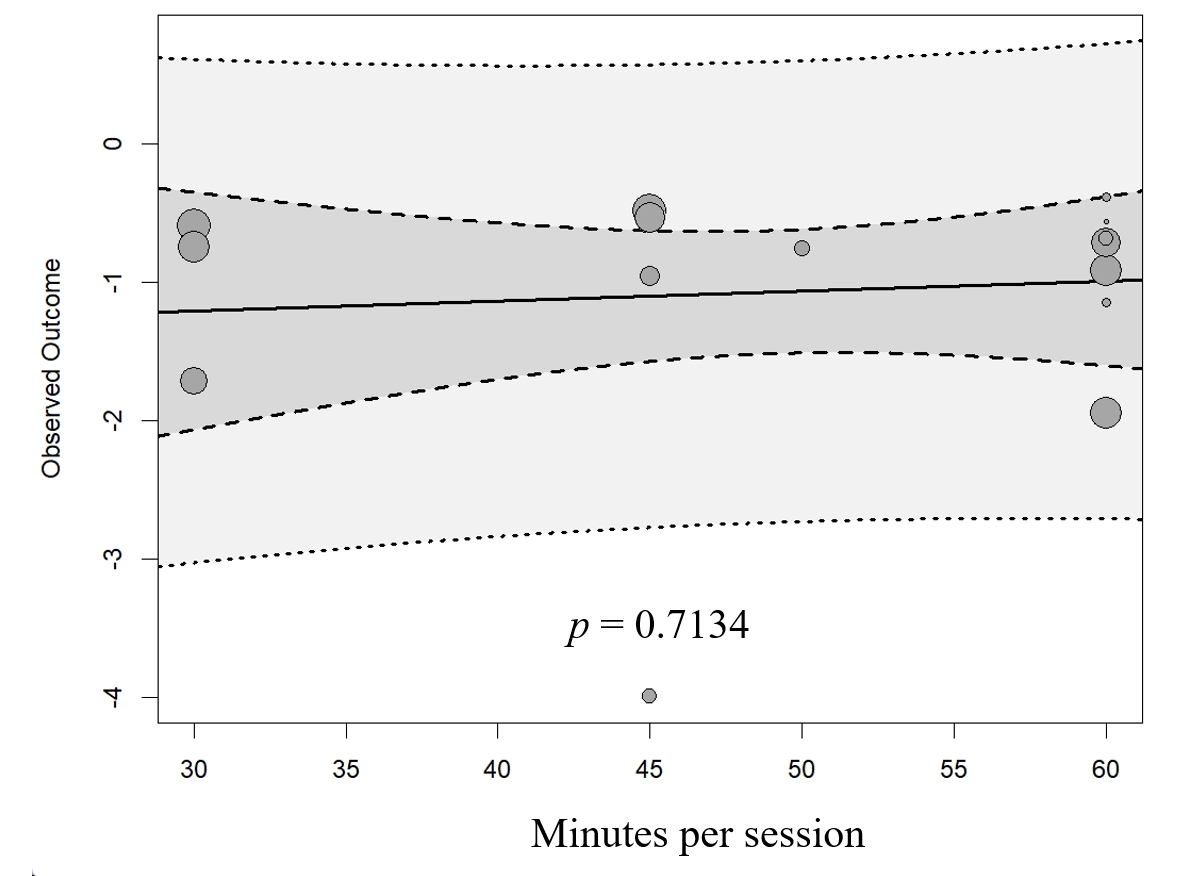
**Figure 4.8 Meta-regression analysis result of Minutes per session.**

# Supplementary Data: Sensitivity Analysis Result


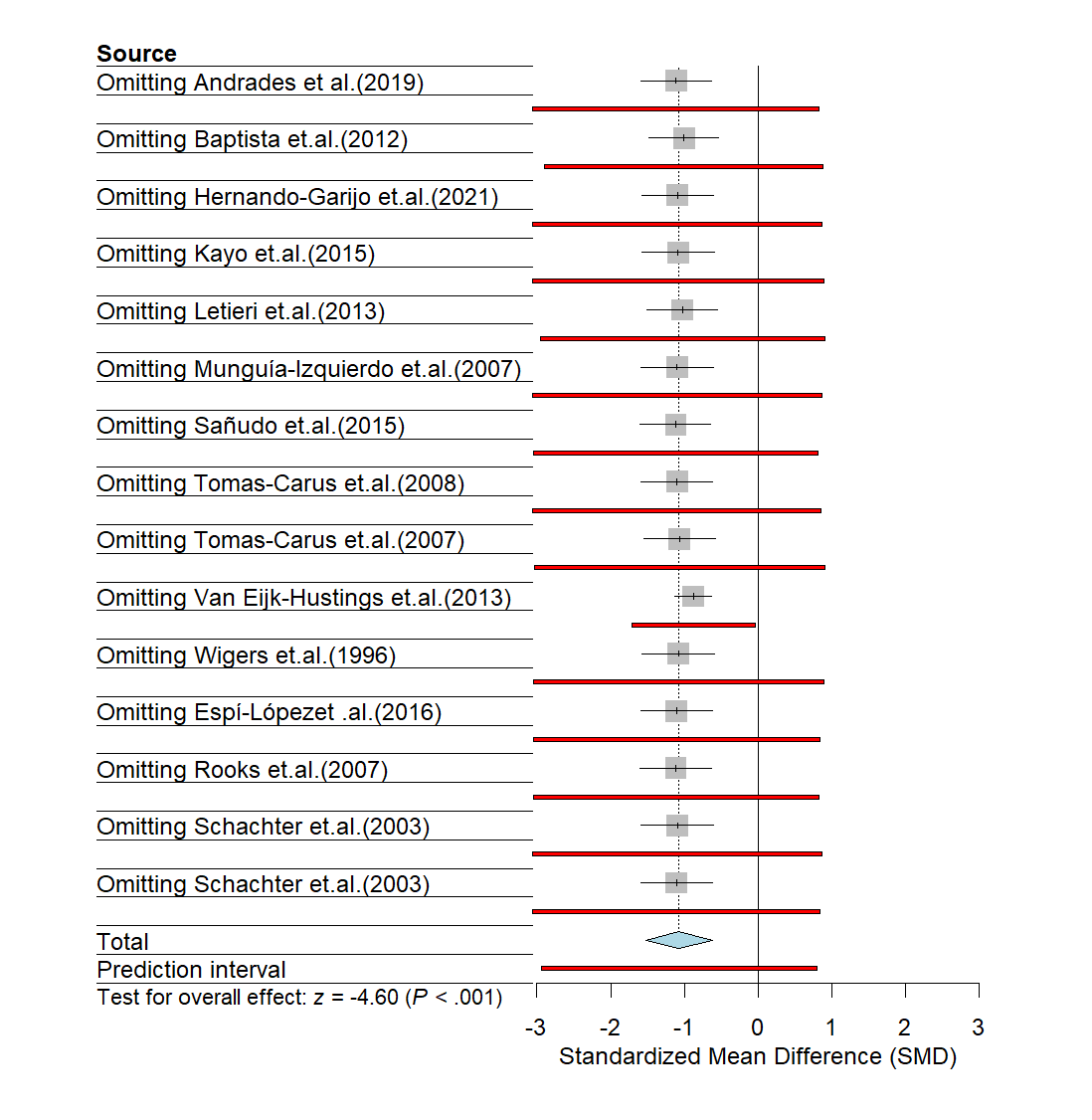


**Figure 8.1 Sensitivity analysis result.**

# Supplementary Data: Network Meta Dose-Response Analysis

We used the MBNMAdose package in R (version 4.2.2, [www.r-project.org](http://www.r-project.org)) and the Bayesian Model-Based Network Meta-Analysis (MBNMA) to summarize the dose-response relationship between aerobic exercise doses and Pain. Connectivity is a key assumption in network meta-dose analysis, and a lack of connectivity may lead to low statistical power and misleading results. By drawing the treatment-level network, we verified the connectivity in this study. We analyzed the data using both the consistency model and the unrelated mean effects model, comparing them in terms of deviance, the number of estimated parameters, and the Deviance Information Criterion (DIC). If DIC values are close, it indicates good consistency in our research.

We assessed transitivity via the MBNMA node-splitting approach. This method splits a particular treatment contrast into direct and indirect evidence and compares their contributions. Comparable effects indicate good transitivity. We compared the random and common effects of various models (Emax, restricted cubic spline (RCS), nonparametric model, and exponential model) on aerobic exercise, evaluating them using the DIC, SD, model parameters, and residuals. Since the RCS model provided a better fit based on our data, we chose the RCS model to fit and analyze our results.

## Connectivity of dose-response

In network meta-analysis (NMA), connectivity is a crucial prerequisite. When connectivity is inadequate, such as the absence of direct comparisons, it may weaken the efficacy of statistical analyses and produce misleading conclusions. ^[1]^ In our study, we visually assessed the connectivity status of the networks for aerobic levels of exercise interventions and the level of task metabolic equivalents consumed through the exercise interventions for a week. The results of the study showed that all networks remained well-connected and did not show any disconnections.


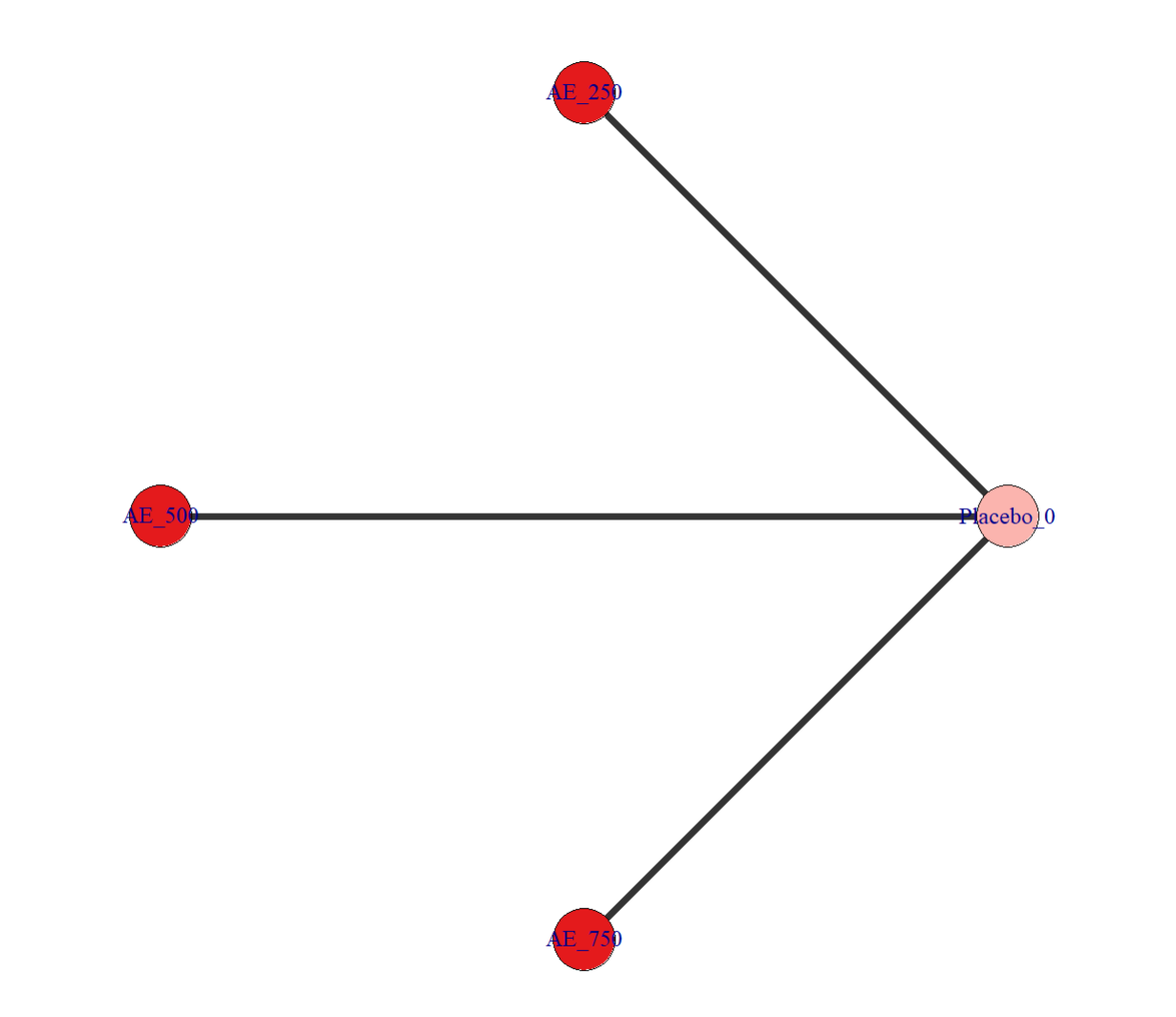


**Figure 6.1.1: Treatment-level network.**


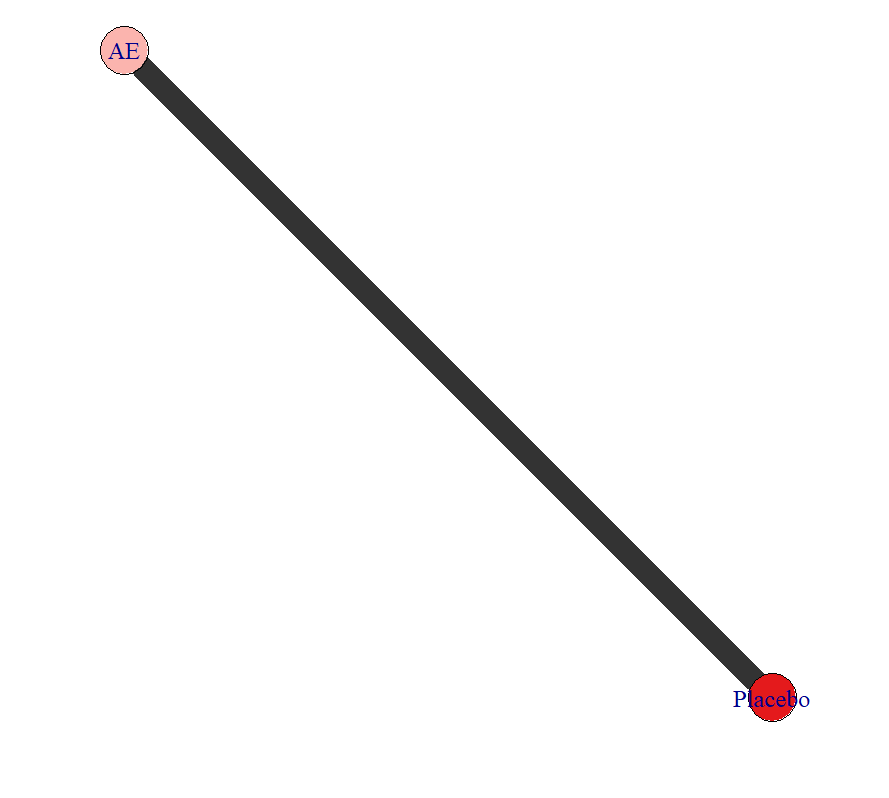


**Figure 6.1.2: Agent-level network.**

**Note:** The first value indicates the specific intervention and the second one is the corresponding dose of that intervention. AE: aerobic exercise; CON: Control group.

## Consistency of dose-response

**Table 6.2.1: Consistent and UME models fit comparison**

| **Model** | **pD** | **Residual deviance** | **Deviance** | **DIC** | **SD** |
| --- | --- | --- | --- | --- | --- |
| **Consistent** | **104.6** | **137.349** | **119.209** | **193.2** | **1.112** |
| **UME** | **29.0** | **139.801** | **121.662** | **192.8** | **1.132** |

**Note:** **pD:** Number of estimated parameters; **DIC:** Deviance Information Criterion; **SD:** Standard Deviation; **UME:** Unrelated Mean Effects. Scientific literature indicated that the main indicator to assess the model fit is the DIC. As lower DIC, better fit.


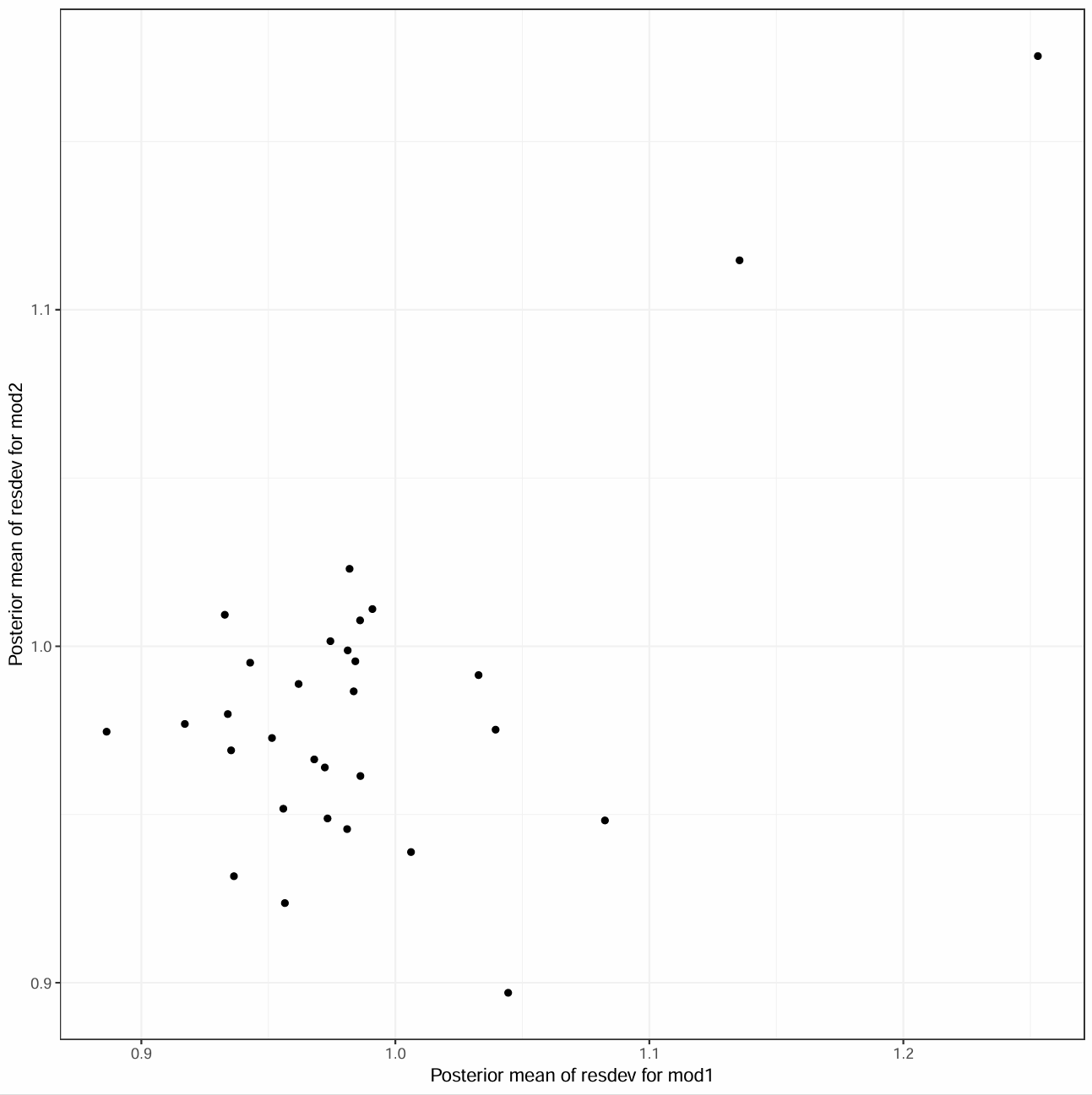


**Figure 6.2.2: Posterior Mean Residual Deviance Comparison Between Two Models.**

**Note:** The Figure shows a scatter plot of the posterior mean residual deviance for two models (mod1 and mod2). The x-axis represents the posterior mean residual deviance from od1, while the y-axis represents the posterior mean residual deviance for mod2. Each dot represents the distribution of residual deviance values for different data points in the two models. Most points are concentrated around 1.0, indicating that the residual deviances of the two models are quite similar for most data points. However, there are a few outliers, suggesting that for some data points, the fitting performance of the two models differs significantly.

## Transitivity of dose-response

We assessed transitivity via the MBNMA node-splitting approach. This method splits and compares contributions for a particular treatment contrast into direct and indirect evidence. Similar effects denote good transitivity. Figure 6.3 below presents the results for transitivity in this meta-analysis.


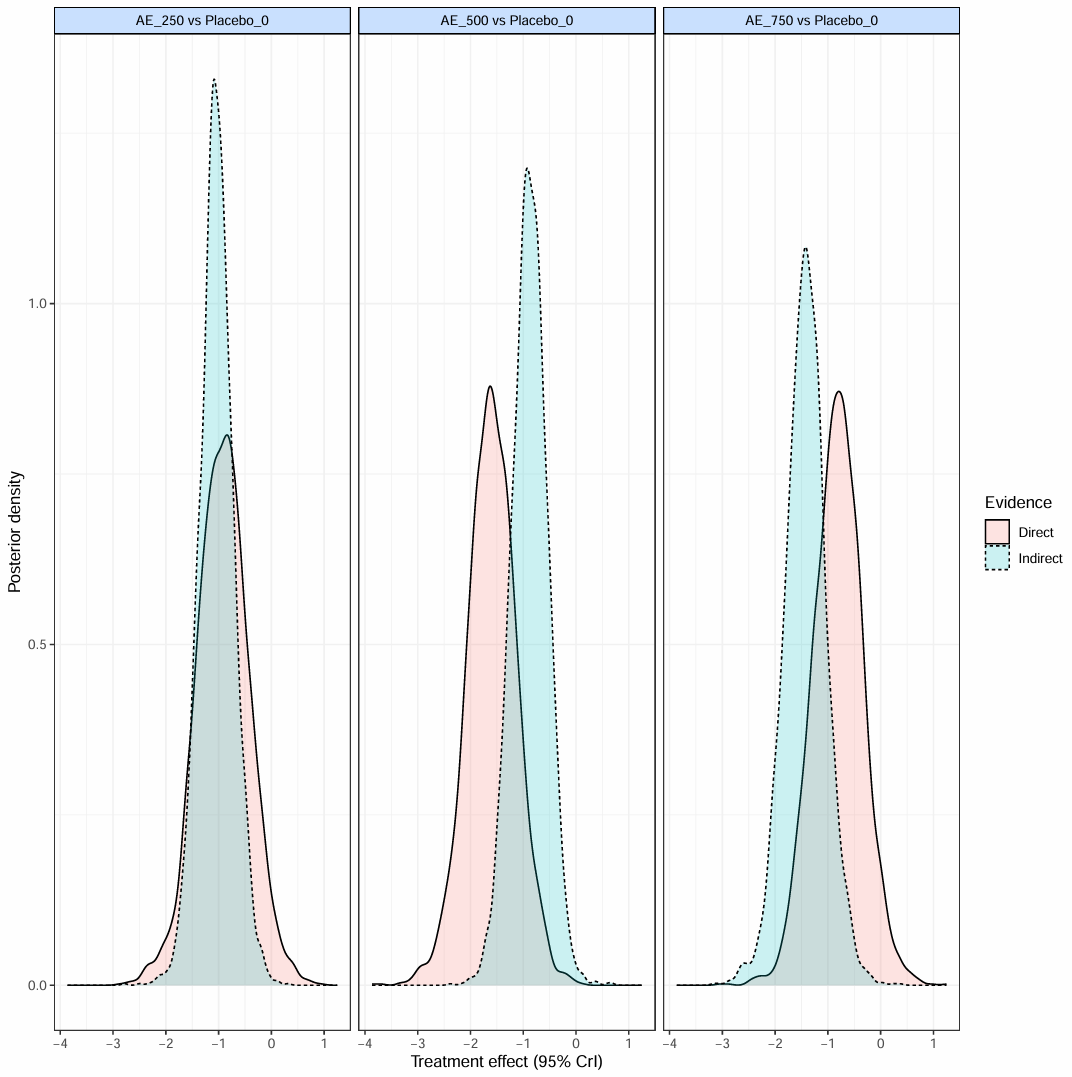


**Figure 6.3: Density Plots of aerobic exercise treatment dose effects.**

# Supplementary Data: Non-Linear Functions and Models Fit Comparison

Figures 7.1 depict the effect size relative to placebo on the link scale for both aerobic and varying doses of physical activity, analyzed under the assumption of independence and unrelatedness among treatments (termed as "split" NMA). The horizontal axis signifies the dose, while the vertical axis indicates the effect size. Each data point signifies the estimated effect size accompanied by its 95% credible interval. This initial step aids in identifying the most appropriate function to fit the data, which can then be applied in a Model-Based Network Meta-Analysis (MBNMA)


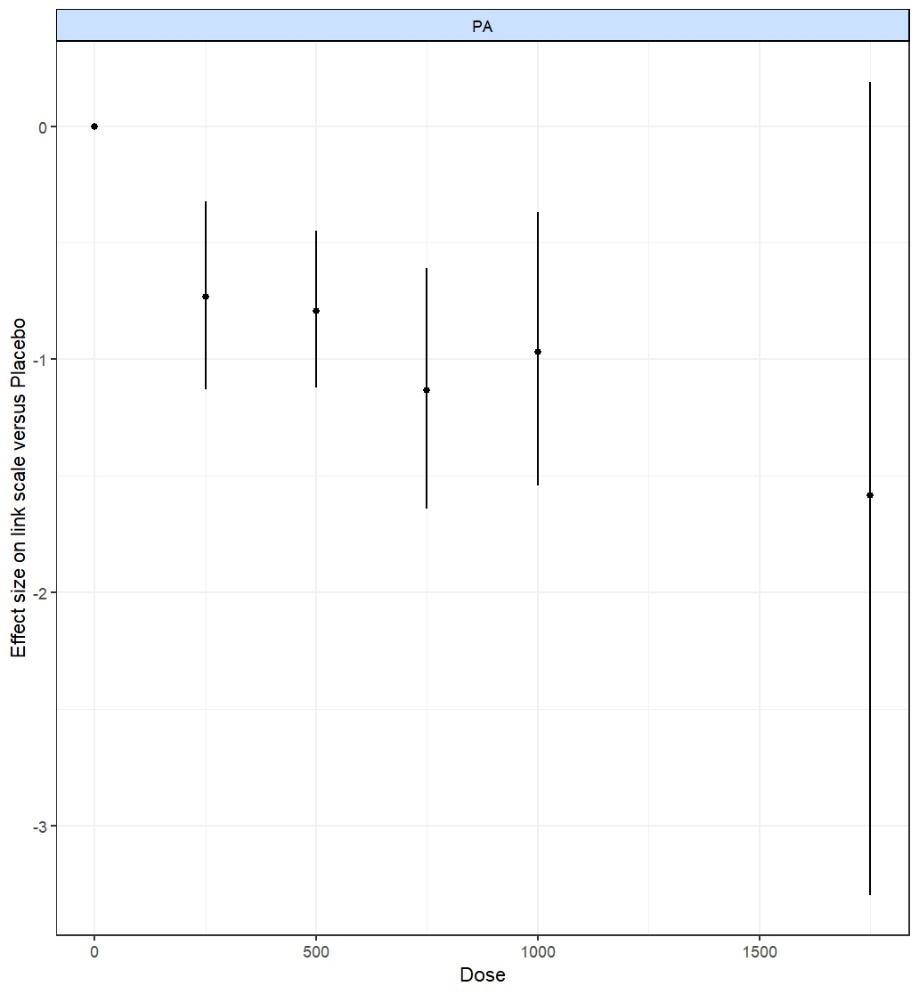


**Figure 7.1: “Split” NMA of aerobic exercise.**

Tables 7.2 show the fit indices for each model applied to the aerobic exercise and the aerobic exercises, respectively. The restricted cubic spline curve provided the best fit in our evaluation and was therefore used in the subsequent analysis.

| **Model** | **DIC** | **SD** | **Deviance** | **Residual deviance** | **pD** |
| --- | --- | --- | --- | --- | --- |
| **Emax (common intervention effects)** | 257.4 | NA | 241.834 | 252.058 | 15.4 |
| **Emax (random intervention effects)** | 47.7 | 0.977 | 19.064 | 29.288 | 28.5 |
| **Restricted cubic spline (common intervention effects; 3knots)** | 75.7 | NA | 59.451 | 69.675 | 16.1 |
| **Restricted cubic spline (random intervention effects; 3knots)** | 40.4 | 0.602 | 16.782 | 6.797 | 23.8 |
| **Non-Parameter (common intervention effects)** | 605.6 | NA | 591.767 | 601.990 | 14.2 |
| **Non-Parameter (random intervention effects)** | 48.8 | 1.833 | 19.487 | 29.711 | 29.3 |
| **Exponential (common intervention)** | 258.9 | NA | 244.111 | 254.334 | 14.9 |
| **Exponential (random Intervention)** | 48.2 | 0.975 | 19.301 | 29.525 | 28.9 |

Figure 7.3: The deviance plot for treatment effects confirms the robustness of our model selection. Further to model fit indices, deviance plots showing the contribution of each data point to the residual deviance are also useful to confirm the robustness of model selection. Each data point should contribute about 1 to the posterior mean deviance, which indicates a good model fit.


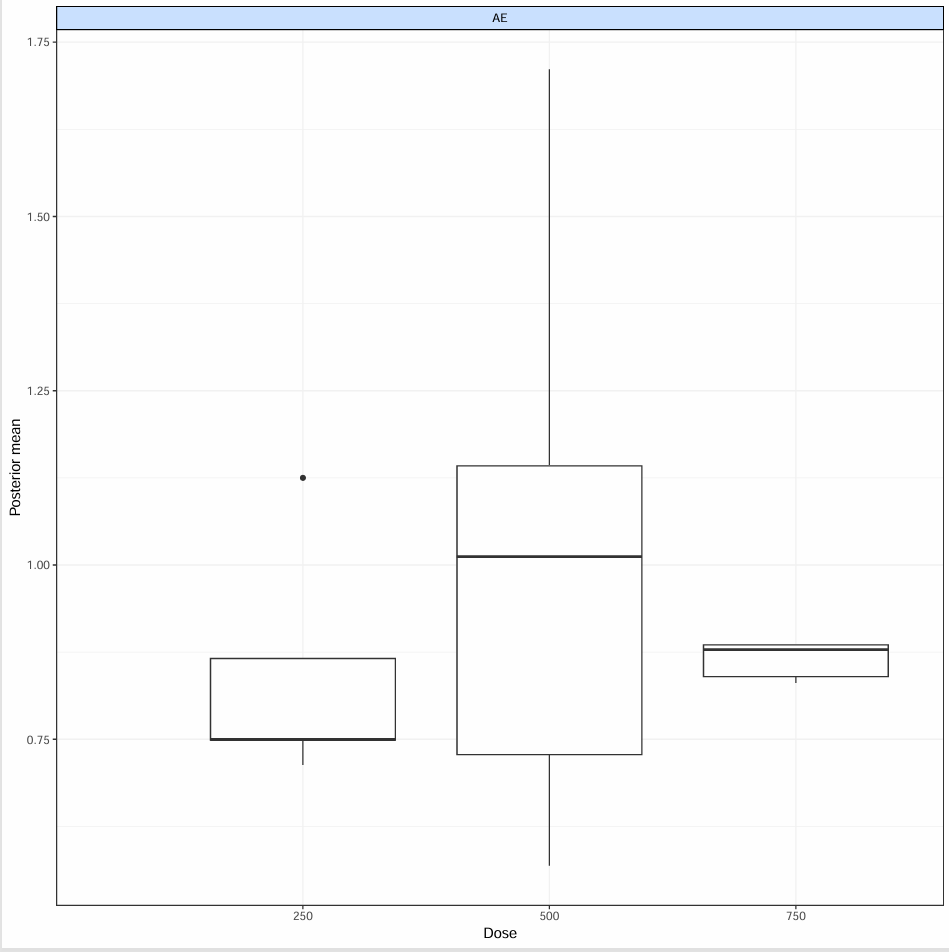


**Figure 7.3: Deviance plot at aerobic exercise level.**

# Supplementary Data: Risk of Bias of Rob2 Assessment


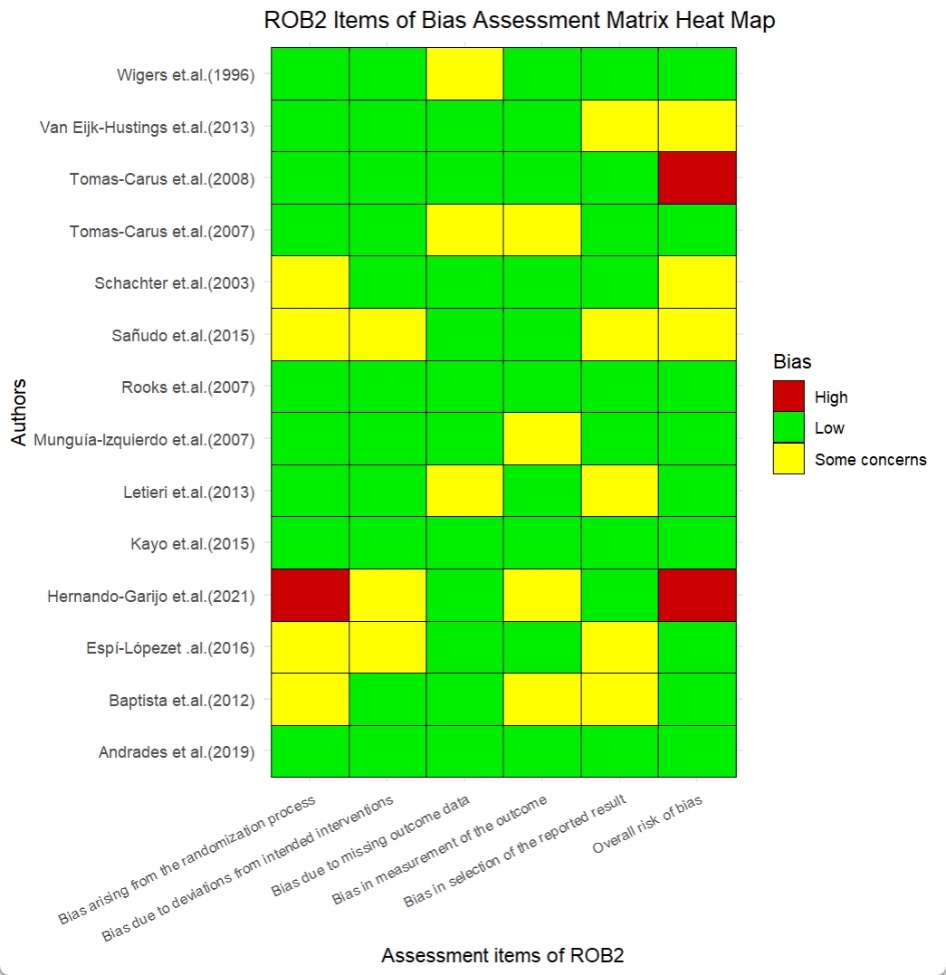


**Figure 8.1 Risk of bias for each included study and domain.**


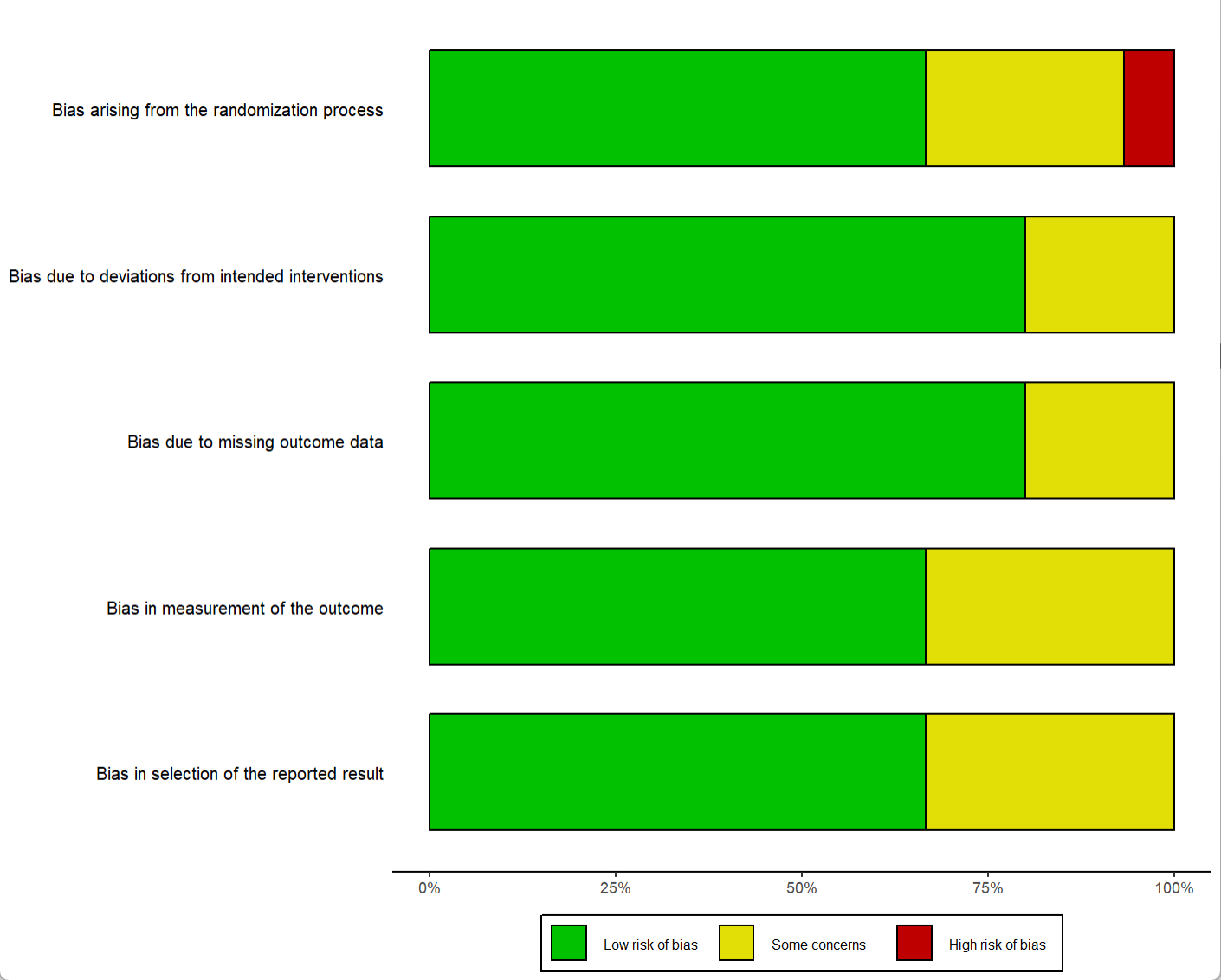


**Figure 8.2: Cochrane Risk of Bias Tool.**

# Supplementary Data: PEDro Scale

Tables 12.1 Methodological assessment of randomized controlled trials included in the systematic review using the PEDro scale.^[5]^

| **Study** | **Pedro-scale-items** | | | | | | | | | | | **Total score** |
| --- | --- | --- | --- | --- | --- | --- | --- | --- | --- | --- | --- | --- |
|  | **Eligibility criteria** | **Random allocation** | **Concealed allocation** | **Baseline comparability** | **Blind subjects** | **Blind therapists** | **Blind assessors** | **Adequate follow-up** | **Intention-to-treat analysis** | **Between-group comparisons** | **Point estimates and variability** |  |
| Andrade et al. (2019) | 1 | 1 | 1 | 1 | 0 | 0 | 0 | 1 | 1 | 1 | 1 | 8 |
| Baptista et.al. (2012) | 1 | 1 | 1 | 1 | 1 | 0 | 0 | 1 | 1 | 1 | 1 | 9 |
| Hernando-Garijo et.al. (2021) | 1 | 1 | 1 | 0 | 0 | 1 | 0 | 1 | 1 | 1 | 1 | 8 |
| Kayo et.al. (2015) | 1 | 1 | 1 | 1 | 0 | 0 | 0 | 1 | 1 | 1 | 1 | 8 |
| Letieri et.al. (2013) | 1 | 1 | 1 | 0 | 0 | 0 | 0 | 1 | 1 | 1 | 1 | 7 |
| Munguía-Izquierdo et.al. (2007) | 1 | 1 | 0 | 1 | 0 | 0 | 0 | 1 | 1 | 1 | 1 | 7 |
| Sañudo et.al. (2015) | 1 | 1 | 0 | 1 | 0 | 0 | 0 | 1 | 1 | 1 | 1 | 7 |
| Tomas-Carus et.al. (2008) | 1 | 1 | 1 | 1 | 0 | 1 | 0 | 1 | 1 | 1 | 1 | 9 |
| Tomas-Carus et.al. (2007) | 1 | 1 | 0 | 1 | 0 | 0 | 1 | 1 | 1 | 1 | 1 | 8 |
| Van Eijk-Hustings et.al. (2013) | 1 | 1 | 1 | 1 | 0 | 0 | 0 | 1 | 1 | 1 | 1 | 8 |
| Wigers et.al. (1996) | 1 | 1 | 1 | 1 | 0 | 0 | 0 | 1 | 1 | 1 | 1 | 8 |
| Espí-López et.al. (2016) | 1 | 1 | 0 | 1 | 0 | 0 | 0 | 1 | 1 | 1 | 1 | 7 |
| Rooks et.al. (2007) | 1 | 0 | 1 | 1 | 0 | 0 | 0 | 1 | 1 | 1 | 1 | 7 |
| Schachter et.al. (2003) | 1 | 1 | 0 | 1 | 0 | 0 | 0 | 1 | 1 | 1 | 1 | 7 |
